# The Traditional Chinese Medicine Compound Hezi Qingyou Formula Controls the MAPK Signaling Pathway for Alleviating Gastric Ulcer Induced by Acetic Acid in Rats

**DOI:** 10.1155/jimr/2178079

**Published:** 2026-01-30

**Authors:** Zhong Feng, Ruixia Wei, Junhao Tian, Hui Li, Haobo Chen, Yajie Hao, Meiyun Chen, Bingmei Su, Yuqian Lai, Mingjin Xun, Guimin Zhang, Meicun Yao

**Affiliations:** ^1^ School of Pharmaceutical Sciences (Shenzhen), Sun Yat-sen University, Shenzhen, China, sysu.edu.cn; ^2^ State Key Laboratory of Integration and Innovation of Classic Formula and Modern Chinese Medicine, Lunan Pharmaceutical Group Co. Ltd., Linyi, China, lunan.com.cn; ^3^ International Pharmaceutical Engineering Laboratory in Shandong Province, Shandong New Time Pharmaceutical Co. Ltd., Linyi, China

**Keywords:** acetic acid, fecal metabolism, gastric ulcer, Hezi Qingyou formula, network pharmacology

## Abstract

In clinical practice, Hezi Qingyou Formula (HZQYF) has been observed to effectively alleviate clinical symptoms associated with gastri ulcers, though its precise mechanism of action remains unclear. To investigate the effects of HZQYF on gastric ulcers, we established a rat model of gastric ulcer and examined its impact on ulcer area, pathological changes, inflammatory cytokines, gastric repair factors, levels of T‐superoxide dismutase (SOD), and malondialdehyde (MDA), as well as metabolites in gastric tissue and feces. The results demonstrated that HZQYF significantly reduced the gastric ulcer area and promoted gastric tissue repair in rats. It downregulated the phosphorylation of P‐P38, P‐JNK1/2, and ERK1/2 proteins in the mitogen‐activated protein kinases (MAPKs) pathway, inhibited pro‐inflammatory cytokines such as interleukin (IL)‐6, IL‐1β, and tumor necrosis factor‐α (TNF‐α), enhanced the expression of gastric repair factors including vascular endothelial growth factor (VEGF), epidermal growth factor (EGF), trefoil factor family 2 (TFF2), and prostaglandin E2 (PGE2), reduced MDA content, and increased the activity of the antioxidant enzyme T‐SOD, thereby suppressing inflammatory responses and ameliorating gastric mucosal damage. Metabolomic studies revealed that HZQYF effectively normalized 40 metabolites in gastric tissue through four key metabolic pathways and 39 metabolites in feces through five key metabolic pathways. Notably, it regulated taurine and hypotaurine metabolism, as well as phenylalanine metabolism, restoring metabolite levels to normal and ameliorating metabolic disorders in diseased rats. In conclusion, HZQYF promotes the healing of experimental gastric ulcers in rats through anti‐inflammatory effects, activation of gastric repair factors, and normalization of gastric tissue metabolites, suggesting its potential therapeutic role in the treatment of gastric ulcers.

## 1. Introduction

Gastric ulcer disease is a condition characterized by environmental factors damaging the digestive tract, leading to injury of the stomach mucosa and, in some cases, involving the submucosa [1–3]. Research indicates that the primary culprits behind gastric ulcers are *Helicobacter pylori* infection and the use of nonsteroidal anti‐inflammatory drugs (NSAIDs) [4–6]. This ailment is a global health concern, impacting individuals worldwide and causing a spectrum of health issues. Currently, approximately 4 million people in different regions are affected by this disease every year [7]. For men, the lifetime frequency is around 11%–14%, while for women, it is 8%–11% [8].

For patients with gastric ulcer disease, proton gastric ulcer diseasemp inhibitors, H2 receptor antagonists, and antacids are now the mainstays of traditional treatment [9]. Furthermore, antibiotics and potassium competitive acid blockers are widely recognized as viable antiulcer treatments [10]. However, these therapeutic regimens are accompanied by a variety of unfavorable adverse effects and increased drug resistance [11]. In addition, it has been found that ulcers recur up to 60%–80% after discontinuation of drugs that inhibit gastric acid secretion [11, 12]. Therefore, it may be difficult to effectively treat gastric ulcer disease with chemical drugs alone.

In clinical practice, we found a Chinese medicine formula, which is called “Hezi Qingyou Formula (HZQYF),” that can effectively treat gastric ulcer disease. The formula is composed of *Syzygium aromaticum* (*L*.) *Merr. & L.M.Perry*, *Terminalia chebula Retz*, and *Ficus simplicissima Lour*. Their weight ratio is 1:3:6. In previous studies, HZQYF had promising in vitro *anti-Helicobacter pylori* activities and demonstrated its possible mechanism of action by down regulating the bacterial adhesion, urease, and flagellar gene expression [13]. Numerous studies have found that *Helicobacter pylori* infection is a significant cause of gastric diseases (gastritis and gastric ulcers). Pharmacological studies have found that *Terminalia chebula Retz* can protect gastrointestinal tract and reduce inflammation [14–16]. In addition, our team’s previous research found that *Terminalia chebula Retz* can inhibit the growth of *Helicobacter pylori* and reduce inflammation [17, 18]. *Ficus simplicissima Lour* provides several benefits, including immune system enhancement, antibacterial characteristics, gastric mucosa protection, microcirculation improvement, and antioxidant activity [19, 20]. *Syzygium aromaticum* (*L*.) *Merr. & L.M.Perry* are traditional Chinese medicine used for the treatment of spleen and stomach diseases, belching, and vomiting [13]. Recent studies have demonstrated the potential of *Syzygium aromaticum* (*L*.) *Merr. & L.M.Perry* to fight bacteria [21], enhance innate immune system function [22], treat gastric ulcers and reduce inflammation [23–25]. Three kinds of Chinese medicine are combined to form HZQYF. HZQYF has the effects of *anti-helicobacter pylori* infection, protection of gastric mucosa, improvement of tissue microcirculation, inhibition of inflammation and anti‐oxidation, which are essential for the treatment of gastric ulcers. Our previous study also found that HZQYF can promote the repair of gastric ulcers, but its pharmacological mechanism is still unclear.

In order to better verify that HZQYF has a significant effect on gastric ulcers, we tried to explore it. The experiment was planned to start with the overall chemical composition of HZQYF and related network pharmacological studies to find the pathway with greater influence, and then verified by Western Blotting and metabolomics. The effects of HZQYF on relevant gastric ulcer indicators (such as ulcer area, interleukin (IL)‐6, IL‐1β, and tumor necrosis factor‐α [TNF‐α]) were studied in rats, so as to better elucidate the role and mechanism of HZQYF in the treatment of gastric diseases.

## 2. Materials and Methods

### 2.1. Network Pharmacology Analysis

#### 2.1.1. Acquisition of the Active Components and Potential Targets of HZQYF

The HZQYF extract contains mainly 31 components and this has been reported study [13]. The potential protein targets of active ingredients were accessed from the Swiss Target Prediction database (http://www.swisstargetprediction.ch/) [26]. Genes with probability >0 were acquired, and the duplicated genes in each compound were then deleted. Finally, nonduplicated predicted targets are obtained.

#### 2.1.2. Collection of Potential Targets Related to Gastric Ulcer

To obtain the gastric ulcer targets, the keyword “gastric ulcer” was searched through three datab [27] and DisGeNET (https://www.disgenet.org/home/) [28]. First, the collected targets data for GeneCards was taken as the median. Second, the target data was integrated with the two additional datasets, and any duplicate entries were eliminated. Consequently, the final target library for gastric ulcer was successfully compiled.

#### 2.1.3. Construction of Venn Diagram and Protein–Protein Interaction (PPI) Network

The Venn diagram was constructed by targets of the drug HZQYF for treating gastric ulcers and gastric ulcer related targets into Venny 2.1.0 (https://bioinfogp.cnb.csic.es/tools/venny/) [29], respectively. Thus, drug‐disease intersectant targets are obtained and possible potential targets are screened. Meanwhile, the potential targets of HZQYF for gastric ulcers were screened. Subsequently, the PPI network of targets of the active ingredients against gastric ulcers was constructed by STRING software [30, 31] version 12.0 (http://string-db.org). The research specie was “*Homo sapiens*.” In conclusion, the TSV file was successfully downloaded and subsequently imported into Cytoscape 3.9.1 software for a comprehensive visual analysis, as can be accessed at [https://cytoscape.org/] (https://cytoscape.org/). To assess the topological features of the network’s nodes, three key metrics were employed: degree, betweenness centrality, and closeness centrality. Furthermore, it is important to note that the color and size of the nodes significantly influence their degree values.

#### 2.1.4. Gene Ontology (GO) and Kyoto Encyclopedia of Genes and Genomes (KEGG) Pathway Enrichment Analyses

The database for annotation, visualization and integrated discovery (DAVID), accessible at [https://david.ncifcrf.gov/](https://david.ncifcrf.gov/), was utilized for conducting KEGG pathway enrichment analysis. The process began by importing the list of drug‐disease intersecting targets into the DAVID database. The analysis was focused on “*Homo sapiens*” to ensure species specificity. A stringent threshold of *p*‐value <0.05 was applied to filter significant results. Following this, the top 20 enrichment items, as determined by their *p*‐values in both GO and KEGG analyses, were selected. These items were then imported into the bioinformatics website at [www.bioinformatics.com.cn](http://www.bioinformatics.com.cn) for visualization. The data were graphically represented as bubble charts to provide a clear and intuitive understanding of the enrichment results.

### 2.2. Drugs and Reagents

The scientific name of *Syzygium aromaticum* (*L*.) *Merr. & L.M.Perry*, *Terminalia chebula Retz*, and *Ficus simplicissima Lour*. Plant names were checked with MPNS (http://mpns.kew.org). The specimen source, identiffcation, and storage of three kinds of Chinese medicine was as briefly descripted in our prior study [13, 17, 18, 21].

Kangfuxinye (KFXY) was a gastric ulcer disease medicine purchased from Sichuan Haoyisheng Panxi Pharmaceutical Co., Ltd. P38 mitogen‐activated protein kinases (P38 MAPKs) antibody, P‐P38 MAPK, extracellular regulated protein kinases (Erk1/2), phosphorylation extracellular regulated protein kinases (P‐Erk1/2), Jun‐amino‐terminal kinase 1/2(JNK1/2), phosphor‐Jun‐amino‐terminal Kinase 1/2(P‐JNK1/2), beta‐actin (Actin) were supplied by Cell Signaling Technology (USA). Anti‐rabbit and mouse antibodies were acquired from CST (USA). TNF‐α, IL‐1β, IL‐6, IL‐10, vascular endothelial growth factor (VEGF), epidermal growth factor (EGF), trefoil factor family 2 (TFF2), prostaglandin E2 (PGE2), malondialdehyde (MDA), and superoxide dismutase (SOD) enzyme‐linked immunosorbent assay (ELISA) kit were obtained from Shanghai Enzyme‐linked Biotechnology Co., Ltd. (China).

### 2.3. Preparation of HZQYF Extract

HZQYF extract was prepared by water extraction method. The preparation and chemical composition analysis of HZQYF dried aqueous extract was the same as described in our previous study [13]. The dried extract was kept at −20°C for storage.

### 2.4. Experimental Animals

Healthy male Sprague‐Dawley rats, weighing between 180 and 200 g, were procured from Beijing HFK Bioscience Co., Ltd., with a certificate number of SCXK (Beijing) 2019–0008. These rats were designated for gastric ulcer disease research and were maintained at the New Drug Pharmacology Center of Lunan Pharmaceutical Co., Ltd.

The study was granted approval by the Ethics Committee for Experimental Animals at the State Key Laboratory of Generic Manufacture Technology of Chinese Traditional Medicine (Approval Number: AN‐IACUC‐2023‐072), ensuring ethical and humane treatment of the animals throughout the research process.

### 2.5. Animal Grouping, Model Establishment and Administration

The rats were randomly assigned to six distinct groups, each comprising 10 individuals. Control group (control), model group (model), HZQYF low‐dose group (HZQYF‐L 0.36 g/kg), HZQYF medium‐dose group (HZQYF‐M 0.71 g/kg), and HZQYF high‐dose group (HZQYF‐H 1.42 g/kg), KFXY group (2.7 mL/kg). The clinical dosage of HZQYF is 30 g/day, with an extraction yield of 26.42%. This experiment adopted a converted dosage of 4.76 g/day. Based on the body surface area conversion principle, three administration dose groups were established: HZQYF‐L received 50% of the clinically equivalent dose, HZQYF‐M was administered the clinically equivalent dose, and HZQYF‐H received twice the clinically equivalent dose.

After fasting and depriving of water for 24 h, the rats were anesthetized with 1% pentobarbital sodium. The abdominal area of the rats was shaved and disinfected, and an approximately 2 cm incision was made with surgical scissors to expose the stomach. The anterior wall of the glandular stomach near the pylorus was identified. A microsyringe was used to inject 20 μL of 30% acetic acid from the serosal surface into the submucosal layer of the gastric mucosa; the control group received an equal volume of physiological saline, and the incision was then sutured.

Drug administration was started on the second postoperative day, and the appropriate drug was given to each group. The control group and the model group were gavage‐fed with equal volumes of sterile water. On the 14th postoperative day fasting without water for 12 h, anesthesia was executed. Blood, stool samples, and gastric of rat were collected for subsequent experiments.

### 2.6. Histopathological Analysis

Rat stomach tissue is fixed in 4% paraformaldehyde. After being removed, it undergoes dehydration and embedding to produce 5 μm paraffin sections, which are then dried at 60°C for 30 min. Deparaffinization is performed by soaking the sections in xylene and various concentrations of ethanol. HE staining is carried out using the Leica st5020 automatic stainer. Ultimately, the stained gastric tissue sections underwent pathological examination using the Nikon TS2 microscope (Nikon Instech in Tokyo, Japan).

### 2.7. Ulcer Index

After ligating the cardia and pylorus with fine sutures, the stomach is removed. Inject 10 mL of physiological saline (2–8°C) into the stomach using a syringe and allow it to remain fixed for 15 min. Open the stomach and rinse the interior with physiological saline (2–8°C) until it is completely clean. Measure the longest and widest dimensions of the ulcer using a vernier caliper, then calculate the ulcer area by multiplying these two values to determine the ulcer index.

### 2.8. ELISA

After the rats were anesthetized, blood was collected from the abdominal cardia vein, and then centrifuged at 1000 × *g* for 15 min at 2–8°C within 30 min of collection. The supernatant is taken to obtain the serum sample. Serum samples were stored at −80°C to avoid repeated freezing and thawing. ELISA kits were utilized to measure the serum concentrations of IL‐1β, IL‐6, IL‐10, MDA, and SOD following the provided guidelines.

The stomach tissue was washed with chilled PBS to eliminate remaining blood, weighed out at 100 mg, and then minced before being mixed with 900 μL of PBS and homogenized on ice. Finally, the homogenate was centrifuged at 5000 × *g* for 20 min and the supernatant was taken for testing. The levels of bFGF, VEGF, EGF, TFF2, and PGE2 in serum were detected by ELISA kits according to the instruction.

### 2.9. Fecal Untargeted Metabolomics Analysis

Sample solution for gastric tissue. First, the sample of gastric tissue prepared previously was defrosted quickly at 37°C. Then, 50 mg of it was weighed accurately and into a 5 mL centrifuge tube, and 1 mL of methanol–acetonitrile–water (2:2:1) was added into the tube to extract compounds and precipitate protein in the sample after vortex‐mixing. Next, 100 µL of the liquid was transferred into a 1.5 mL centrifuge tube and vortex‐mixed. After a 5 min still standing in ice‐bath, the mixture in a 1.5 mL centrifuge tube was vortex‐mixed for 10 min and treated with ultrasound for 10 min subsequently. Finally, it was centrifuged at 12,000 rpm/min for 10 min, and supernatant was collected.

Sample solution for feces: First, the sample of feces prepared previously was defrosted quickly at 37°C. Then, 200 mg of it was weighed accurately and Gastric ulcer diseaset into a 10 mL centrifuge tube, and 4 mL of methanol–water (3:1) was added into the tube to extract compounds in the sample after vortex‐mixing. Next, 100 µL of the liquid was transferred into a 1.5 mL centrifuge tube and vortex‐mixed. After a 5 min still standing in ice‐bath, the mixture in a 1.5 mL centrifuge tube was vortex‐mixed for 10 min and treated with ultrasound for 10 min subsequently. Finally, it was centrifuged at 12,000 rpm/min for 10 min, and the supernatant was collected.

A Waters ACQUITY UPLC HSS T3 (2.1 mm × 100 mm, 1.8 μm) column was used as the solid phase. Water containing 5 mM ammonium acetate and 5 mM acetic acid was mobile Phase A, acetonitrile was mobile Phase B. Gradient elution was as follows: 0–0.7 min (flow rate 0.35 mL/min), 1% B; 0.7–9.5 min (flow rate was from 0.35 mL/min to 0.5 mL/min), 1%–99% B; 11.8 min (flow rate 0.5 mL/min), 99% B; 12.1–14.6 min (flow rate 0.5 mL/min), 1% B; 14.8–15.0 min (flow rate 0.35 mL/min), 1% B. Column temperature was 40°C. Injection volume of the sample was 2 µL.

The mass spectrum was performed in Q Exactive Orbitrap mass spectrometer by both positive and negative polarity. The scan range was from 70 to 1050 *m*/*z*. Acquisition time was 0–30 min. The ion source was heated electrospray ionization source (HESI) at a temperature of 350°C. Spray voltage in positive polarity was 4 kV and in negative polarity was 3.5 kV. The capillary temperature was at 325°C and the vaporizer temperature was at 300°C. High purity nitrogen was used as sheath and auxiliary gas. The sheath gas flow rate was 40 Arb, and the auxiliary gas flow rate was 20 Arb. Scan mode was Full MS/dd‐MS2. Resolution of Full MS and dd‐MS2 were 7000 and 17,500 separately.

A variety of analytical techniques were employed to obtain the results, encompassing data‐dependent acquisition methods such as ddMS2 and parent ion list‐MS2, Fourier transform infrared spectroscopy (FTIR, Full Scan, with a resolution of 30,000), and additional methodologies. The metabolic variations among the three groups were elucidated through the application of orthogonal partial least squares‐discriminant analysis (OPLS‐DA) and partial least squares discriminant analysis (PLS‐DA). The associated metabolic pathways were investigated based on the database, accessed through the Metabolomic Analyst platform. (https://www.metaboanalyst.ca/faces/home.xhtml).

Western Blotting Homogenize rat gastric tissue using RIPA lysis buffer and lyse it on ice for 30 min. After centrifuging the samples at 12,000 rpm for 20 min to collect the supernatant, measure the protein concentration and dilute the samples to equalize their concentrations. Then, subject equal volumes of protein samples (10 μL) to electrophoresis at 120 V for 90 min, followed by transfer onto a PVDF membrane. Block non‐specific binding by incubating the membrane in 5% skim milk at room temperature for 90 min. Add the primary antibody and incubate overnight at 4°C, then add the secondary antibody and incubate at room temperature for 60 min. After washing, apply the luminescent solution and perform chemiluminescence detection to capture and save the image. Finally, use Image J software for quantitative analysis of the images.

### 2.10. Statistical Analysis

Quantitative data were presented as the mean ± standard deviation (SD) and were analyzed utilizing SPSS 19.0 software. The GraphPad Prism 8.0 was utilized for the data analysis. Differences between groups were compared by one‐way ANOVA and Tukey’s multiple comparison test. A *p*‐value of less than 0.05 was set as the threshold for statistical significance.

## 3. Results

### 3.1. Exploration of the Mechanism of Action of HZQYF on the Gastric Ulcer Through Network Pharmacology Analysis

#### 3.1.1. Ingredients Screening and Gastric Ulcer Diseasetative Targets Prediction of HZQYF

Referring to previous studies, 2003 targets were obtained using 31 of the identified components in HZQYF using the method of Peng et al. [21]. After the process of deduplication, a total of 226 unique targets for HZQYF were identified.

### 3.2. Screening of Gastric Ulcer Targets

Utilizing the keyword “gastric ulcer,” a total of 1445 disease targets were extracted from gene cards after median filtering of the obtained disease targets. Concurrently, 559 and 136 disease targets were retrieved from OMIM and DisGeNET, respectively. By integrating the disease targets from these three databases and removing duplicates, a comprehensive list of 1940 disease targets was compiled. Furthermore, 226 targets associated with HZQYF and the 1940 disease‐related targets were imported into Venny 2.1.0 to create a Venn diagram (Figure 1A). Through this analysis, 99 drug targets were identified as overlapping with the disease targets, which are considered potential therapeutic targets for HZQYF in the treatment of gastric ulcer.

Figure 1Network pharmacology analysis results of the impact of HZQYF on gastric ulcer disease. (A) The Venn diagram showed the targets of HZQYF and gastric ulcer. (B) The protein–protein interaction network, the higher the degree value, the larger and darker the node. (C) GO enrichment results of the potential targets, the ordinate is −log_10_ (*p*‐value). (D) KEGG pathway enrichment results of the potential targets. The abscissa represents gene ratio, the size of the dots indicates the gene count, and the color of the dots stands for the −log_10_ (*p*‐value).

**Figure:**
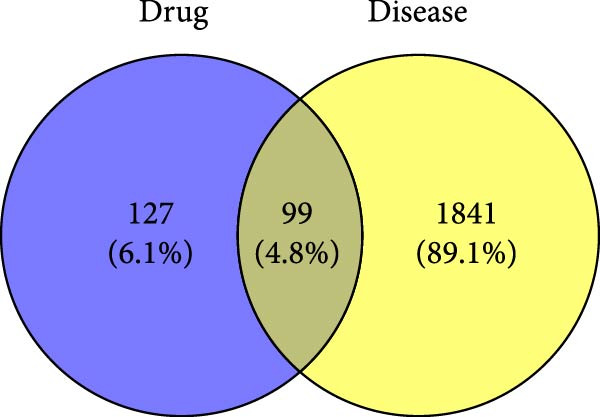
(A)

**Figure:**
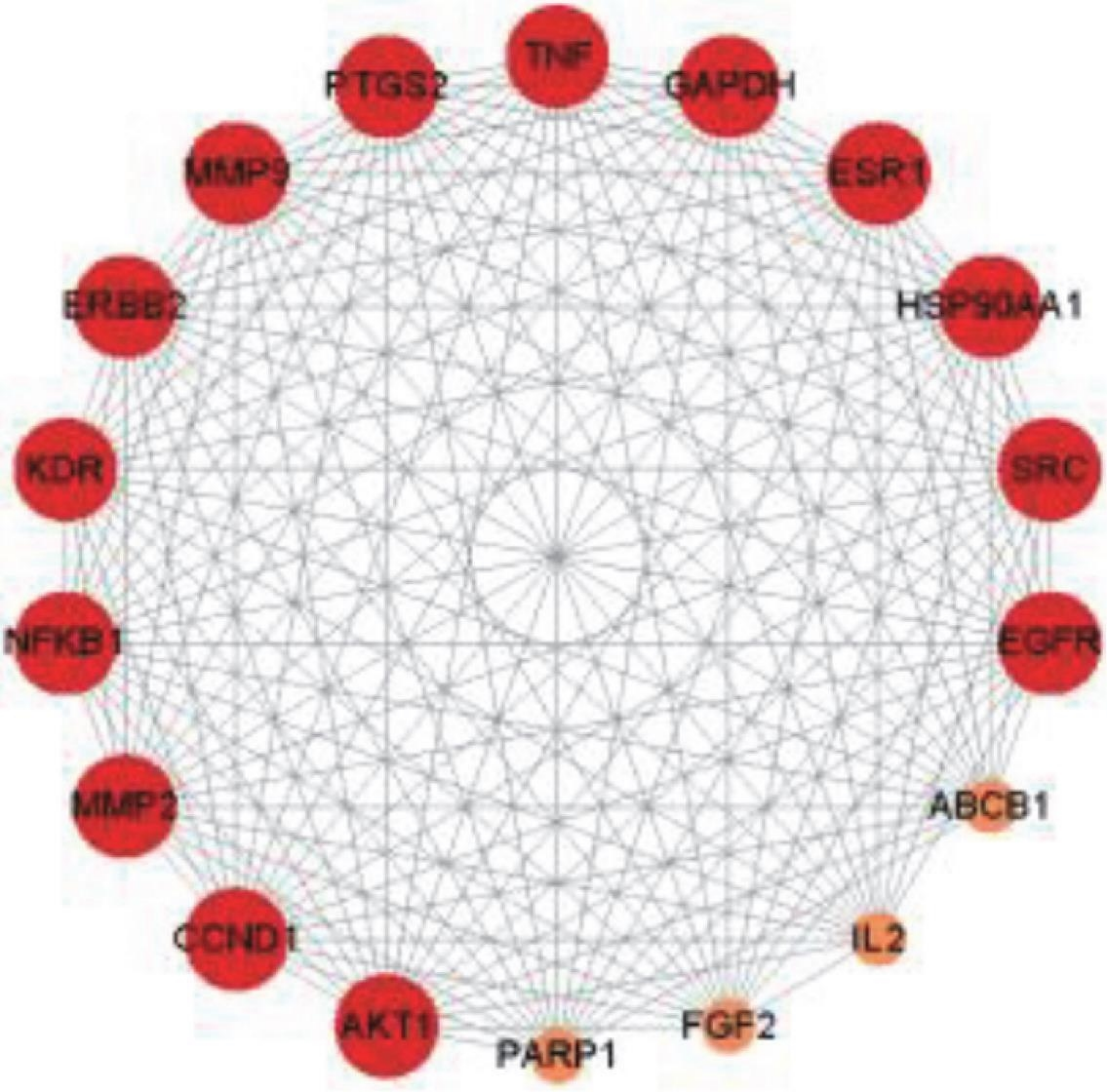
(B)

**Figure:**
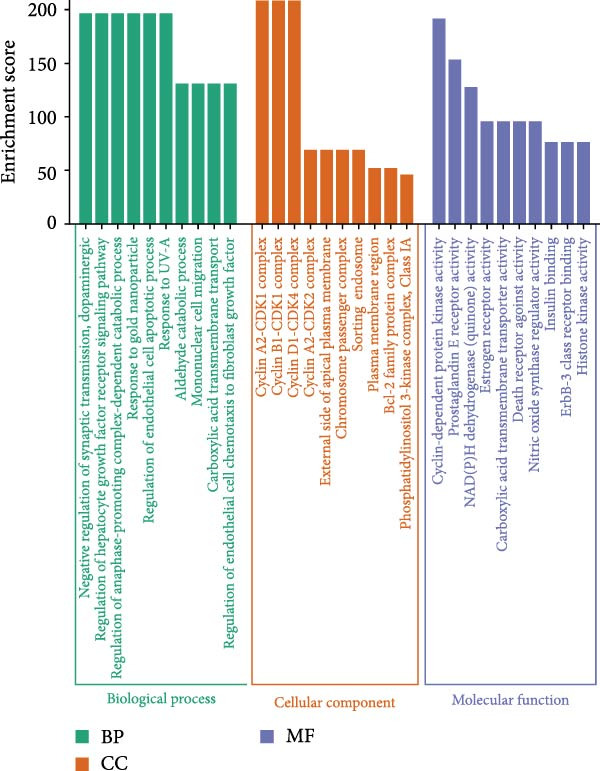
(C)

**Figure:**
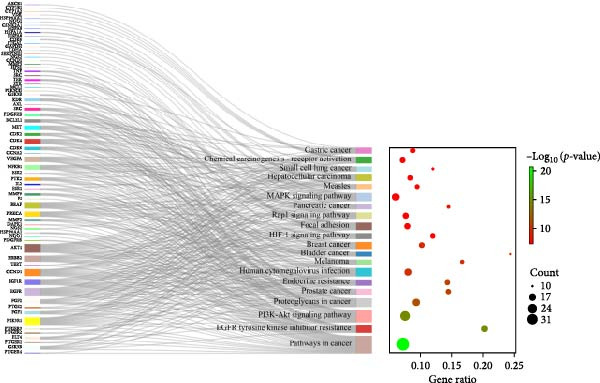
(D)

### 3.3. Identification of the Core Targets and the Main Active Components of HZQYF Against Gastric Ulcer

The 99 potential targets of HZQYF were integrated into the STRING database to map out a PPI network. By employing Cytoscape 3.9.1, we visualized the network, focusing on nodes with high degree values to uncover central targets. The PPI network we obtained included 18 nodes and 149 edges. The nodes’ size and color intensity were directly related to their degree values, which signified their level of connectivity and importance. Within this network, 14 proteins with significant degree values were pinpointed as central, including STAT3, MAPK3, and several other key proteins, as illustrated in Figure 1D.

### 3.4. GO and KEGG Pathway Enrichment Analysis

To delve into the roles of HZQYF components in treating gastric ulcers, GO and KEGG pathway enrichment analyses were conducted on the 99 potential targets of HZQYF using the DAVID website. A total of 582 GO terms were significantly enriched, comprising 408 BP, 68 cellular component CC, and 106 MF terms. The top 10 significantly enriched terms for BP, CC, and MF, ranked by their *p*‐values, are depicted in Figure 1C.

The primary BP terms encompassed negative regulation of synaptic transmission, dopaminergic signaling, regulation of hepatocyte growth factor receptor signaling pathway, regulation of anaphase–promoting complex‐dependent catabolic process, response to gold nanoparticle, and regulation of endothelial cell apoptotic process (Figure 1C). The predominant CC terms included cyclin A2‐CDK1 complex, cyclin B1‐CDK1 complex, and cyclin D1‐CDK4 complex (Figure 1C). The key MF terms involved cyclin‐dependent protein kinase activity, prostaglandin E receptor activity, and NAD(P)H dehydrogenase (quinone) activity (Figure 1C).

Furthermore, KEGG pathway enrichment analysis was conducted to uncover the signaling pathways influenced by HZQYF in the treatment of gastric ulcers. A total of 133 pathway entries were enriched, with the top 20 pathways illustrated (Figure 1D). Among these pathways, the MAPK signaling pathways were identified as being associated with the inflammatory response.

### 3.5. Protective Effect of HZQYF on Rats

As shown in Figure 2, the structural layers of the gastric tissue of rats in the control group were intact and clearly visible, with no local inflammatory infiltration or tissue edema; the cells of the upper layer of the gastric mucosa were neatly arranged, with intact cellular structure, with no cellular defects, detachment, or exudation of inflammatory substances; and the submucosal layer was free of edema and inflammatory cellular infiltration (Figure 2A). In the model group, the gastric tissue of rats was incomplete in structure, with defects, detachment and the cellular structure of the submucosa was obviously edematous, the structure was swollen, and hemorrhagic spots could be seen locally, accompanied by a large number of inflammatory cell infiltration (Figure 2B). In the HZQYF‐L and HZQYF‐M groups, the gastric mucous membrane of the rats was more complete and the submucosal layer was slightly edematous (Figure 2C,D), while in the HZQYF‐L and HZQYF‐M groups, the mucous membrane layer had a small amount of cellular defects and was repaired to a certain extent, and there was a certain degree of infiltration of inflammatory cells in the submucosal layer, and the gastric tissues of the rats in the HZQYF‐H group were more complete, with the submucosal layer being slightly edematous, and a large number of inflammatory cells disappearing from the infiltration (Figure 2E). The gastric tissue structure of rats in the KFXY group was repaired completely, edema was slightly observed in the submucosa, and a large number of inflammatory cells were disappeared (Figure 2F).

Figure 2Effects of HZQYF on pathological changes of the gastric tissue in rats, representative pictures of the colon tissue pathology results of each group (200x). Scale bar = 50 μm. (A) is the control group; (B) is the model group; (C) is the HZQYF‐L group; (D) is the HZQYF‐M group; (E) is the HZYQF‐H group; (F) is the KFXY group.

**Figure:**
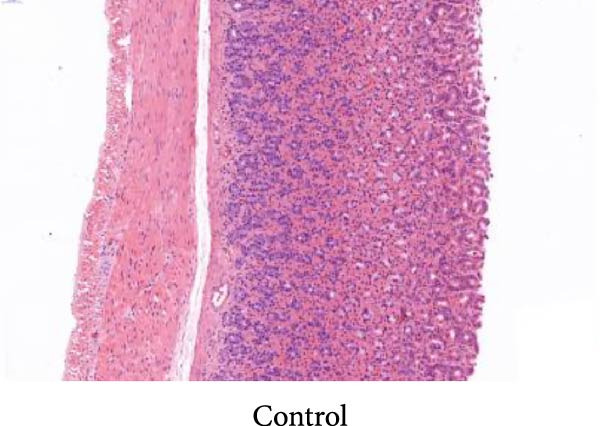
(A)

**Figure:**
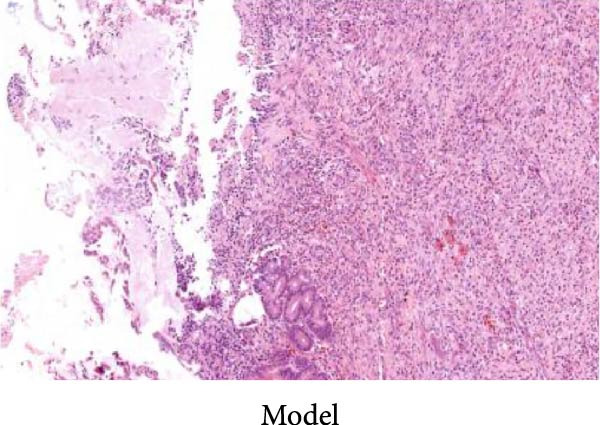
(B)

**Figure:**
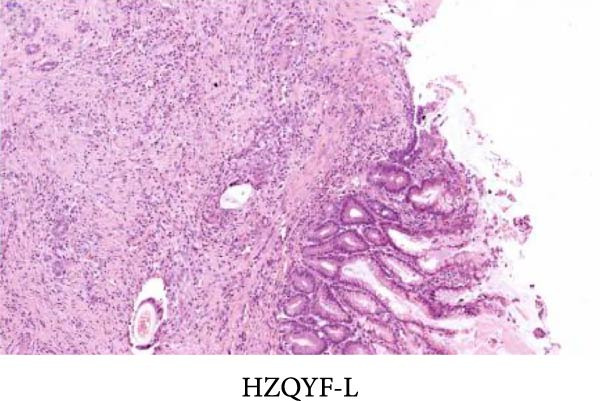
(C)

**Figure:**
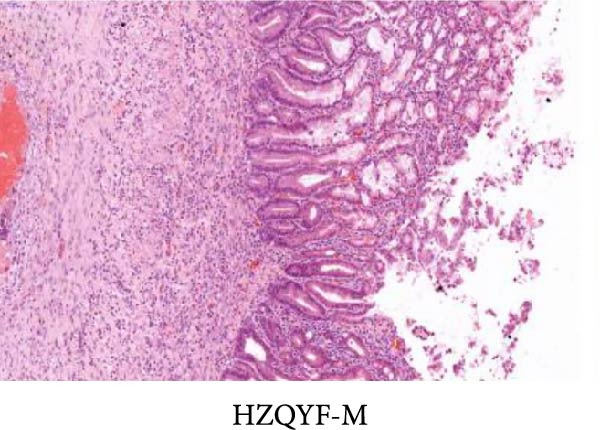
(D)

**Figure:**
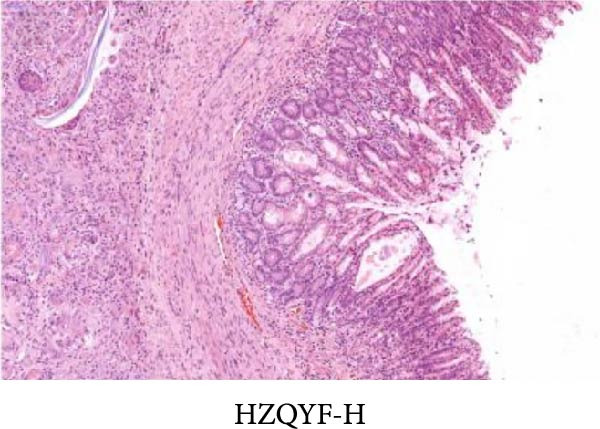
(E)

**Figure:**
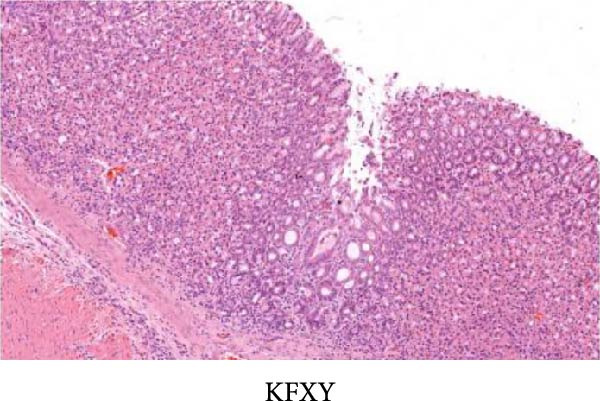
(F)

As can be seen from Figure 2, compared with the Control group, the HZQYF group showed different degrees of reduction in ulcer area, with a statistically significant difference (*p* < 0.05). The positive control group (KFXY group) also had a significant reduction in ulcer area (*p* < 0.05).

### 3.6. HZQYF Alleviated Inflammation in Rats

As depicted in Figures 3B–D, the model group rats exhibited increased serum levels of IL‐6, IL‐1β, and TNF‐α, suggesting a significant inflammatory reaction. However, the oral administration of HZQYF markedly suppressed the secretion of these serum inflammatory factors (*p* < 0.05), suggesting a significant anti‐inflammatory effect of HZQYF.

Figure 3Effect of HZQYF on ulcer area (A) and serum inflammatory factors in rat (*n* = 10). Serum IL‐6 (B), IL‐1β (C), and TNF‐α (D) were assayed by ELISA. Compared with control group,  ^∗^ *p* < 0.05,  ^∗∗^ *p* < 0.01; compared with the model group, ^#^ *p* < 0.05, ^##^ *p* < 0.01.

**Figure:**
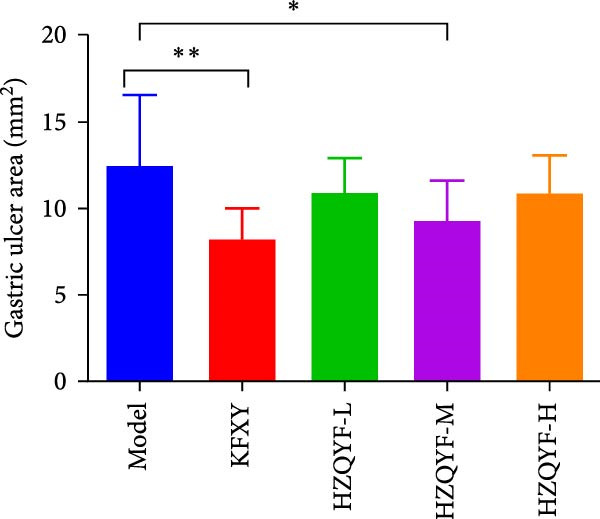
(A)

**Figure:**
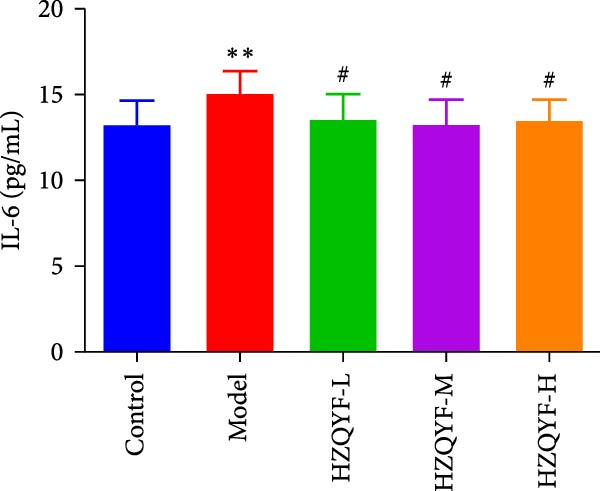
(B)

**Figure:**
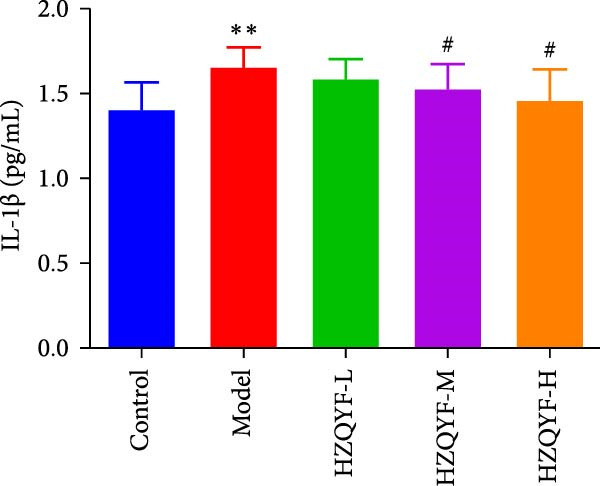
(C)

**Figure:**
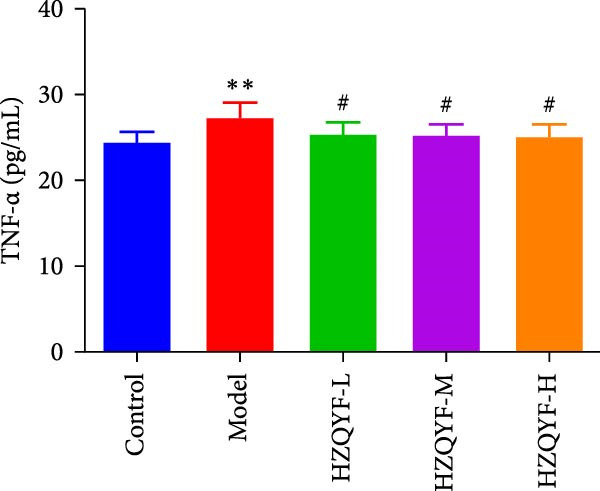
(D)

### 3.7. Effects on Serum VEGF, EGF, TFF2, and PGE2 in Rats

As illustrated in Figure 4, the serum VEGF levels in the Model group were markedly higher than those in the control group (*p* < 0.05), but no significant variation was observed between the HZQYF group and the model group, as shown in Figure 4A. In contrast to both the control and model groups, the serum EGF content in rats across all HZQYF dose groups exhibited a tendency towards an increase, as depicted in Figure 4B. In comparison to the control group, the model group had significantly lower serum levels of PGE2 and TFF2 (*p* < 0.05), as shown in Figure 4C,D. The HZQYF group, however, showed a significant increase in these levels compared to the Model group (*p* < 0.05). These findings suggest that HZQYF may have a regulatory effect on these biomarkers, potentially contributing to its therapeutic impact.

Figure 4Serum VEGF (A), EGF (B), TFF2 (C), PGE2 (D), MDA (E), and SOD (F) were assayed by ELISA (*n* = 10). Compared with control group,  ^∗^ *p* < 0.05,  ^∗∗^ *p* < 0.01; compared with the model group, ^#^ *p* < 0.05, ^##^ *p* < 0.01.

**Figure:**
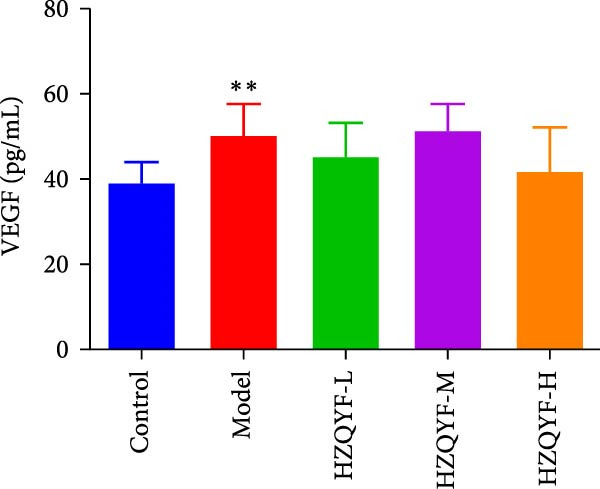
(A)

**Figure:**
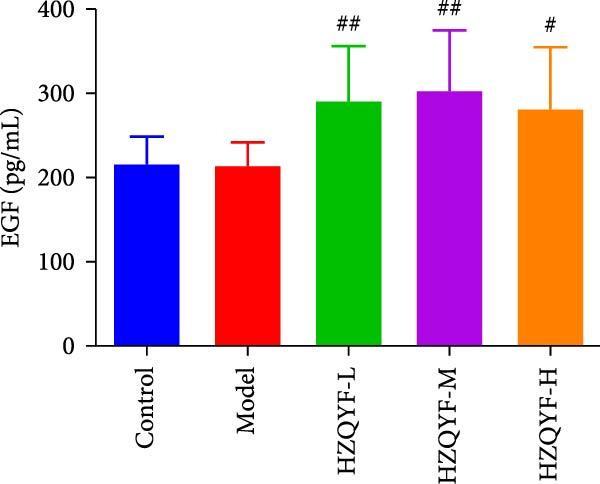
(B)

**Figure:**
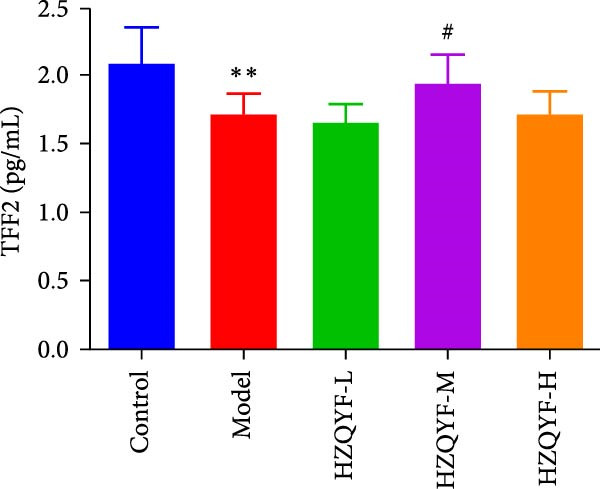
(C)

**Figure:**
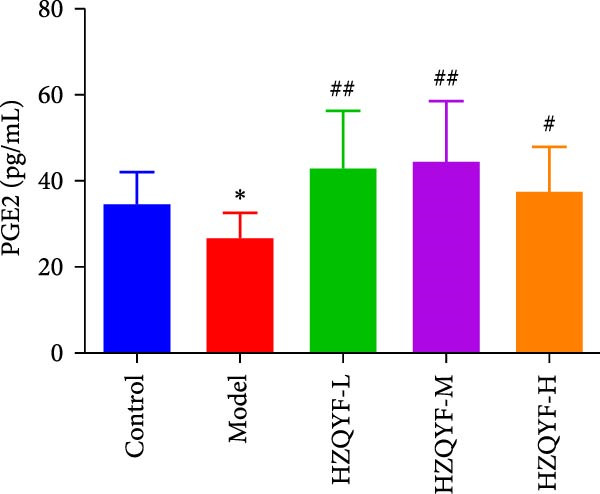
(D)

**Figure:**
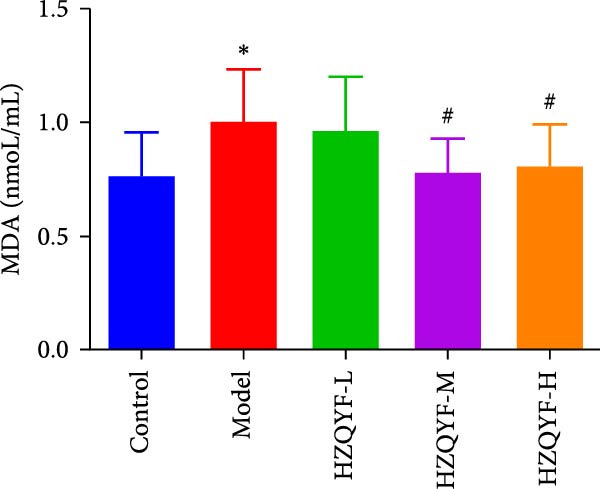
(E)

**Figure:**
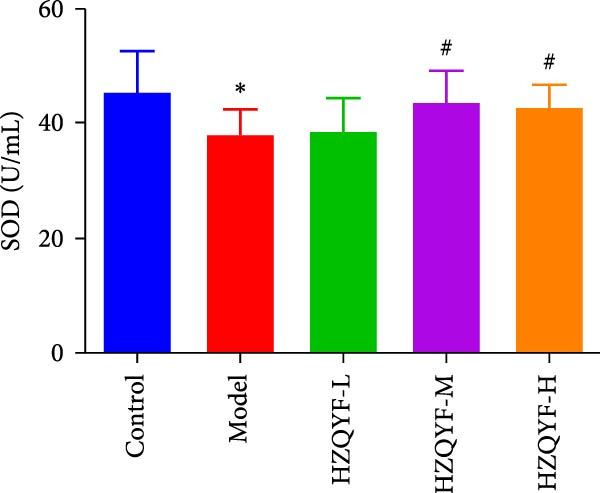
(F)

### 3.8. Effects on SOD and MDA in Serum of Rats

As depicted in Figure 4E, compared to the control group, the concentration of MDA in the serum of the model group was significantly increased (*p* < 0.05). However, the HZQYF group exhibited a reduction in MDA content compared to the model group, which was also statistically significant (*p* < 0.05). Figure 4F illustrates that the SOD content in the serum of rats in the model group was significantly decreased compared to the control group (*p* < 0.05). In contrast, the serum SOD levels in the HZQYF groups were significantly elevated compared to the model group (*p* < 0.05). These findings suggest that HZQYF may play a role in modulating oxidative stress markers, potentially mitigating oxidative damage in the rats. By reducing MDA levels and enhancing SOD activity, HZQYF may contribute to antioxidant defense, protecting cells from damage. These results provide novel insights into the mechanisms by which HZQYF may exert its therapeutic effects in the treatment of gastric ulcers.

### 3.9. HZQYF Regulates the Abnormalities of Metabolites in Rat Gastric Tissues

To reveal the metabolic changes in rat gastric tissues, gastric tissue samples were analyzed from three groups of rats (*n* = 8), control group, model group and HZQYF‐M group, using LC‐MS/MS. 3484 metabolites were identified in the gastric tissues. The three groups of rats were separated by PLS‐DA (Figure 5A) and OPLS‐DA (Figure 5B,D) and clustered in each group, which showed significant differences among the three groups of rats. The R2 and Q2 of the OPLS‐DA scoring analysis verified that the predictability was high and the reliability was good (Figure 5C,E). Differential metabolites were identified by screening (VIP > 1.0, *p*  < 0.05). A total of 40 common metabolites were obtained from the three groups (Table 1). The apparent changes in metabolites in the three groups were compared by heat map analysis (Figure 5F). There was a significant increase in 29 metabolites and a decrease in 11 metabolites in the model group compared to the control group. The expression of these metabolites was reversed after oral administration of HZQYF. Differentially expressed metabolites were further enriched using the MetaboAnalyst open database to generate KEGG metabolic pathways. Five different metabolic pathways were significantly enriched, including taurine and hypotaurine metabolism, lysine degradation, pyrimidine metabolism, drug metabolism‐cytochrome P450, and gastric ulcer disease serine metabolism (Figure 5G). Notably, taurine and low taurine metabolism (*p* < 0.026) was selected as the major metabolic pathway. HMDB0000965 was identified to be related to taurine and low taurine metabolism. MFTPA0502 was involved in lysine degradation. HMDB0000296 was mainly involved in pyrimidine metabolism. HMDB0015084 was mainly involved in drug metabolism‐cytochrome P450. HMDB0000157 is involved in Gastric ulcer disease serine metabolism. As shown in Figure 4H–L, compared with the Control group, the levels of MFTPA0502 were increased in the Model group, while the levels of HMDB0000965, HMDB0000296, HMDB0015084 and HMDB0000157 were decreased. Surprisingly, the expression trends of the above metabolisms were strongly reversed after administration of HZQYF, especially HMDB0000965, MFTPA0502 and HMDB0015084 (*p* < 0.05). In conclusion, HZQYF can effectively restore metabolites in the gastric tissue of rats, thus contributing to the repair of gastric ulcers.

Figure 5Effect of HZQYF on gastric metabolic dysregulation in rats (*n* = 8). PLS‐DA (A) and OPLS‐DA (B, D) analysis score plots of metabolic profiles in the ion mode. (C, E) Cross‐validation results in ion mode. (F) Heat map showing differences in metabolite abundance. (G) KEGG pathway analysis of differential metabolites in the three groups. (H–L) Abundance comparison of key metabolites among the five groups based on KEGG results. Compared with control group,  ^∗^ *p* < 0.05,  ^∗∗^ *p* < 0.01; compared with the model group, ^#^ *p* < 0.05, ^##^ *p* < 0.01.

**Figure:**
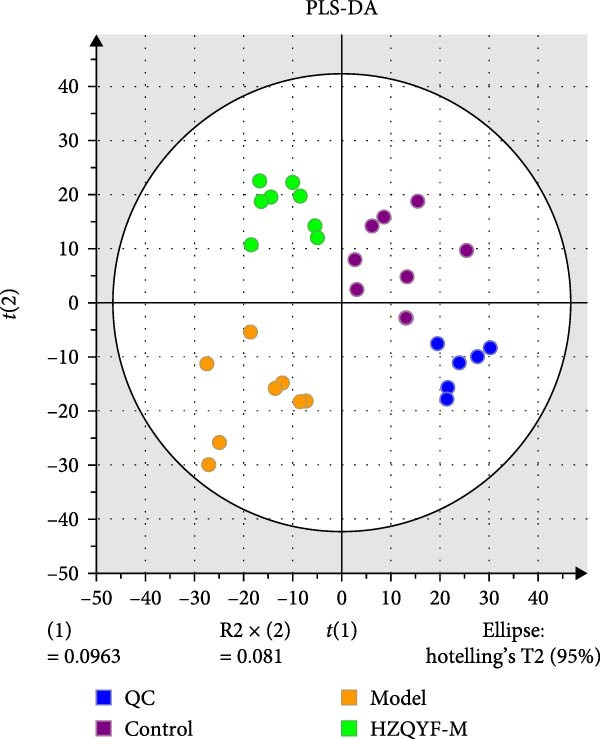
(A)

**Figure:**
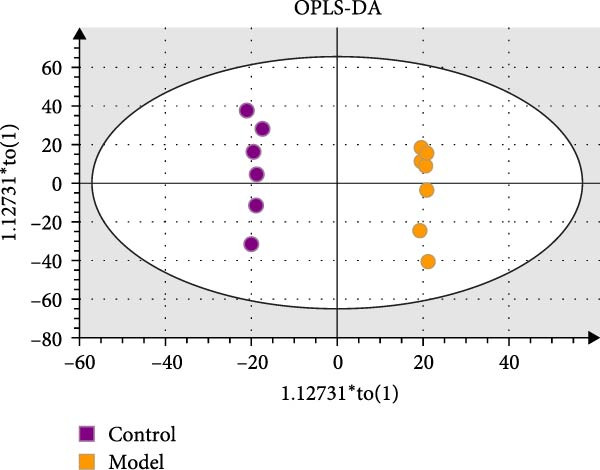
(B)

**Figure:**
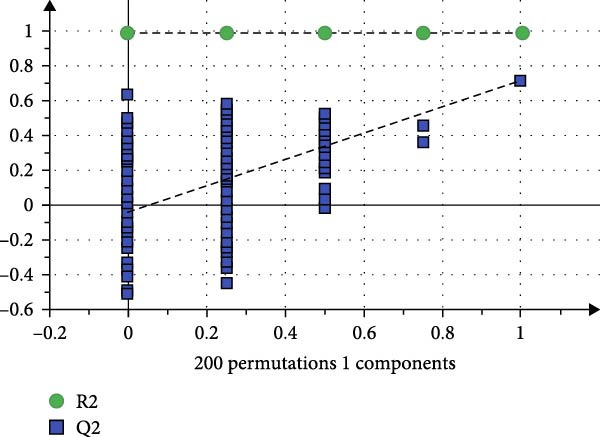
(C)

**Figure:**
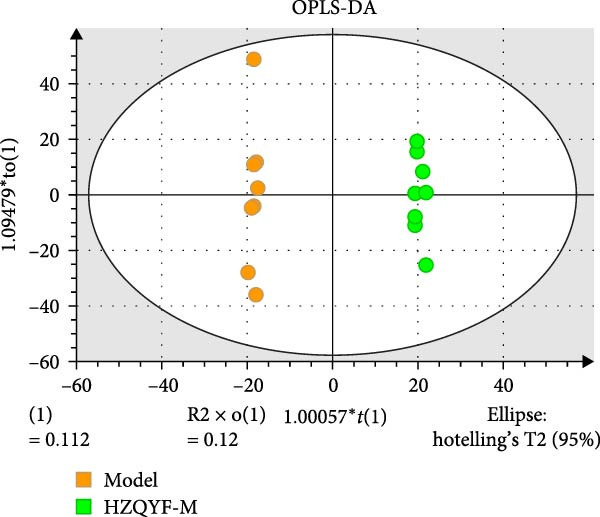
(D)

**Figure:**
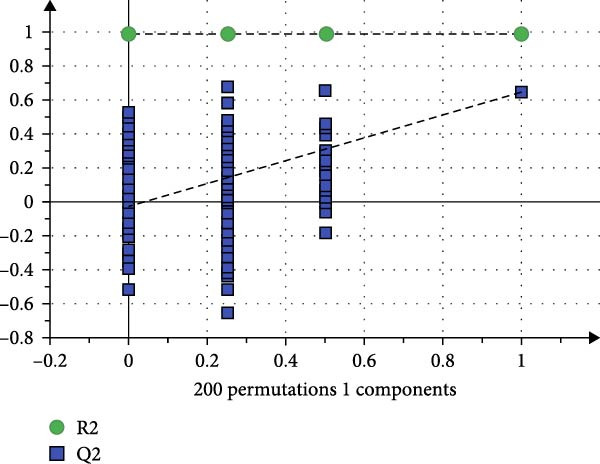
(E)

**Figure:**
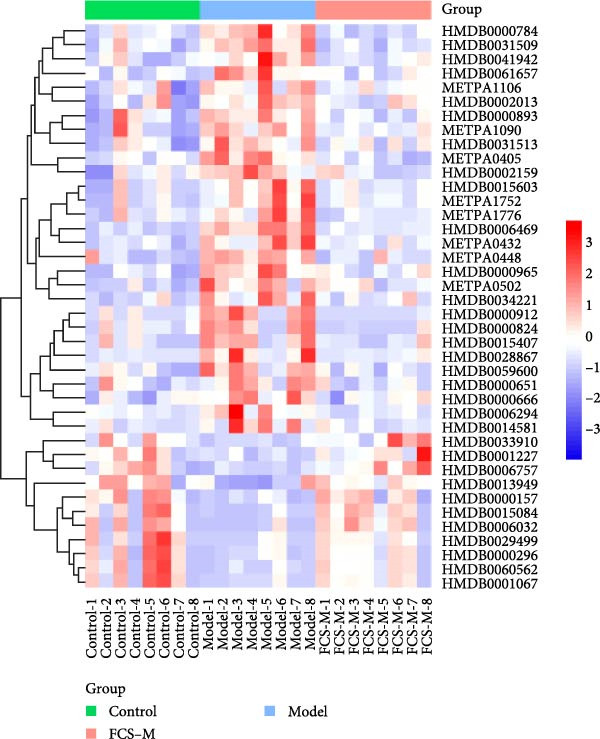
(F)

**Figure:**
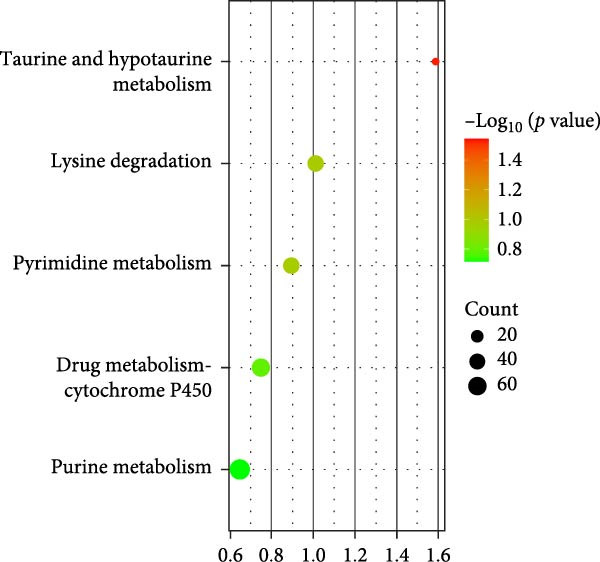
(G)

**Figure:**
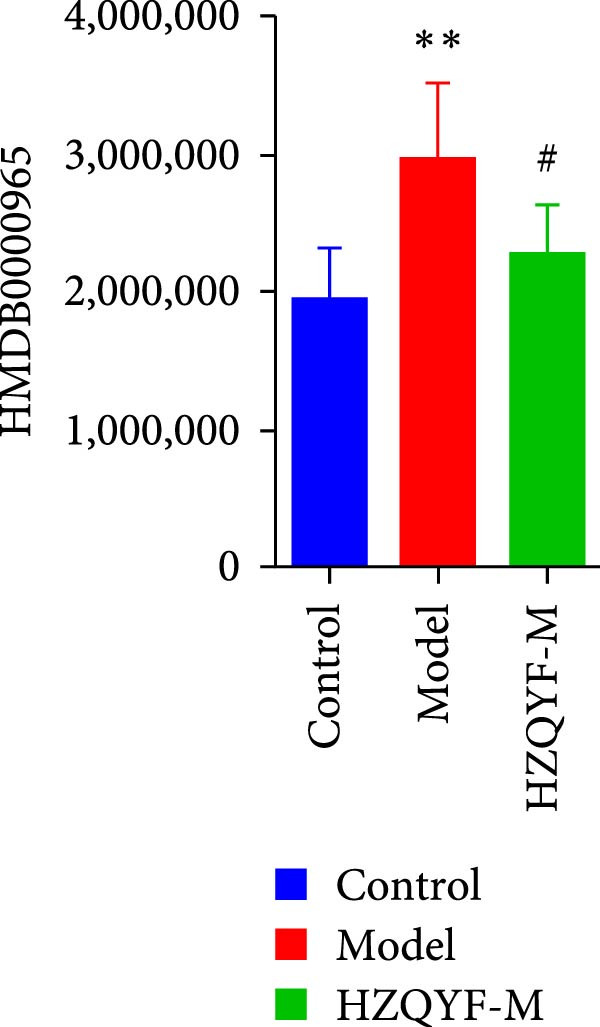
(H)

**Figure:**
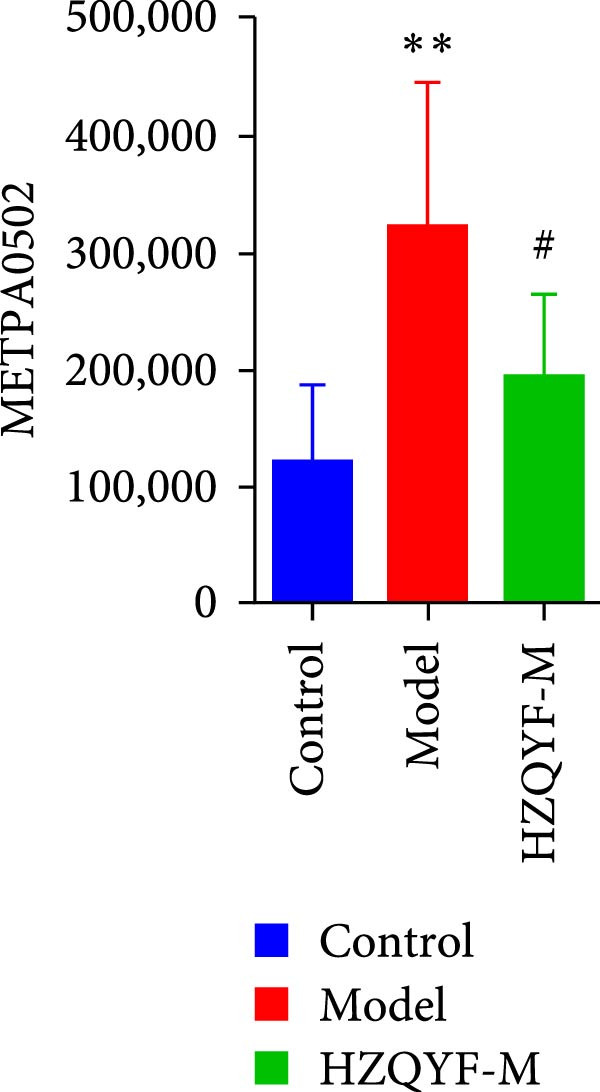
(I)

**Figure:**
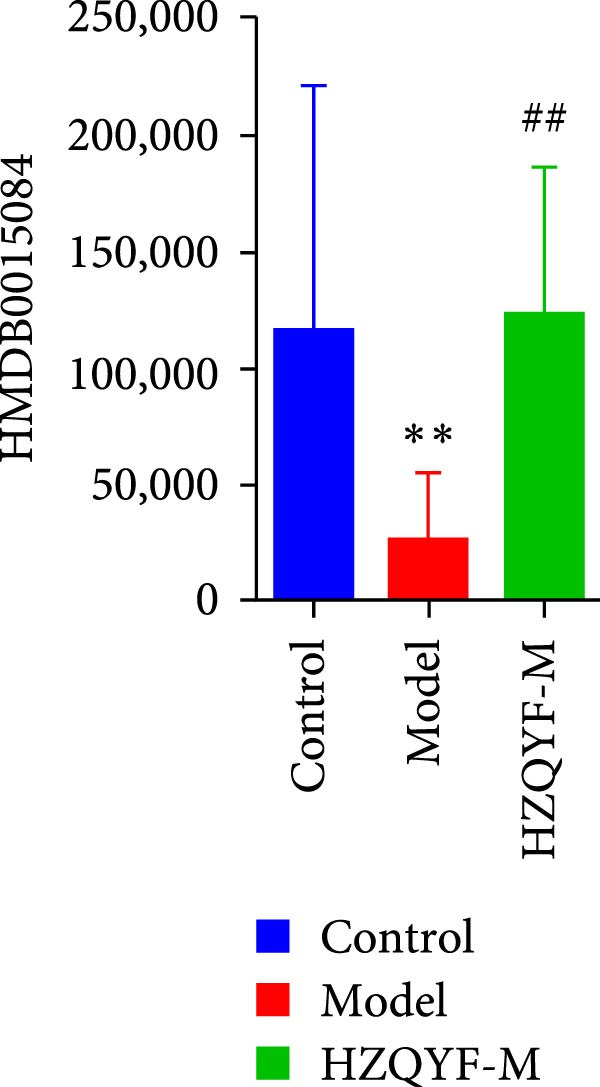
(J)

**Figure:**
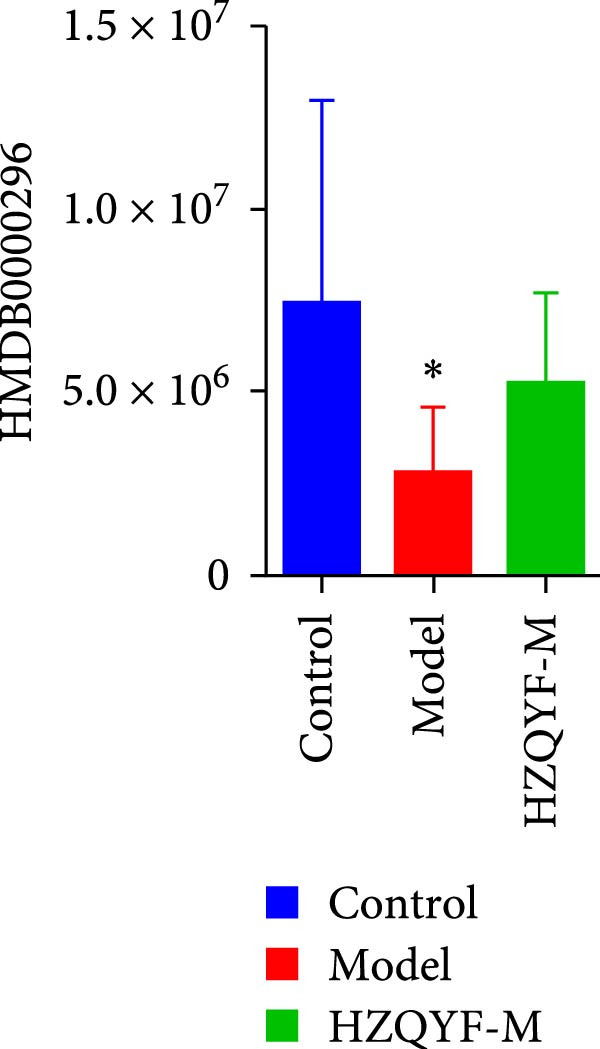
(K)

**Figure:**
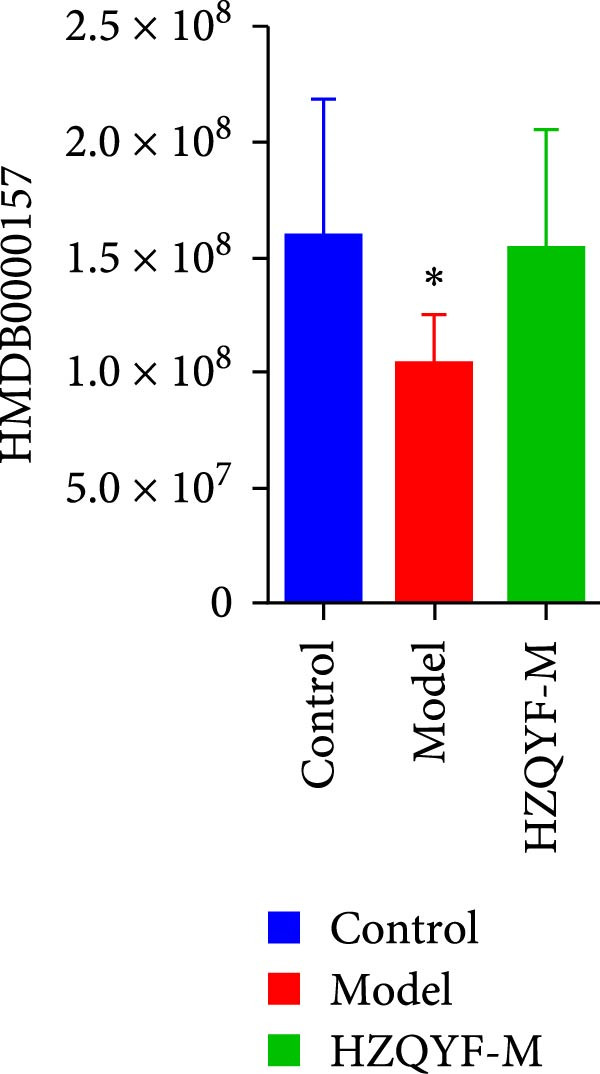
(L)

**Table 1:** Potential biomarkers in gastric tissue of rats with gastric ulcer treated with HZQYF.

Number	Name	Formula	RT (min)	Ion mode	HMDB	Model vs. control	HZQYF‐M vs. model
1	8‐methylthiooctanaldoxime	C9H19NOS	4.929	ESI‐	METPA1776	↑ ^∗^	↓##
2	2‐oxo‐10‐methylthiodecanoic acid	C11H20O3S	9.66	ESI‐	METPA1752	↑ ^∗^	↓#
3	N2‐Acetyl‐L‐aminoadipyl‐delta‐phosphate	C8H14NO8P	0.766	ESI‐	METPA1106	↑ ^∗^	↓#
4	5‐Hydroxyconiferaldehyde	C10H10O4	4.251	ESI‐	METPA1090	↑ ^∗^	↓##
5	Procollagen5‐(D‐galactosyloxy)‐L‐lysine	C12H24N2O8	0.732	ESI‐	METPA0502	↑ ^∗∗^	↓#
6	(2R_4S)‐2_4‐Diaminopentanoate	C5H12N2O2	11.467	ESI‐	METPA0448	↑ ^∗∗^	↓##
7	(S)‐4‐Amino‐5‐oxopentanoate	C5H9NO3	0.797	ESI‐	METPA0432	↑ ^∗∗^	↓#
8	S‐(5‐Deoxy‐beta‐D‐ribos‐5‐yl)‐L‐homocysteine	C9H17NO6S	0.901	ESI‐	METPA0405	↑ ^∗∗^	↓##
9	3‐Hydroxypentadecanoic acid	C15H30O3	7.335	ESI‐	HMDB0061657	↑ ^∗^	↓#
10	Captopril‐L‐cysteine	C12H20N2O5S2	1.683	ESI‐	HMDB0060562	↓ ^∗^	↑#
11	5‐Phosphonooxy‐L‐lysine	C6H15N2O6P	0.952	ESI‐	HMDB0059600	↑ ^∗^	↓##
12	AMCC	C7H12N2O4S	1.264	ESI‐	HMDB0041942	↑ ^∗^	↓#
13	(−)‐Quebrachitol	C7H14O6	0.871	ESI‐	HMDB0034221	↑ ^∗∗^	↓#
14	Acetophenone	C8H8O	2.796	ESI‐	HMDB0033910	↓ ^∗^	↑#
15	3‐Hydroxynonanoic acid	C9H18O3	4.689	ESI‐	HMDB0031513	↑ ^∗^	↓#
16	Ethyl(R)‐3‐hydroxyhexanoate	C8H16O3	4.281	ESI‐	HMDB0031509	↑ ^∗∗^	↓##
17	Fukinolic acid	C20H18O11	1.707	ESI‐	HMDB0029499	↓ ^∗^	↑#
m18	4‐Hydroxyprolylleucine	C11H20N2O4	1.183	ESI‐	HMDB0028867	↑ ^∗^	↓#
19	Ximelagatran	C24H35N5O5	9.653	ESI‐	HMDB0015603	↑ ^∗^	↓#
20	Pirbuterol	C12H20N2O3	1.909	ESI‐	HMDB0015407	↑ ^∗^	↓#
21	Felbamate	C11H14N2O4	2.198	ESI‐	HMDB0015084	↓ ^∗^	↑##
22	Allo Gastric ulcer diseaserinol	C5H4N4O	0.921	ESI‐	HMDB0014581	↑ ^∗^	↓#
23	O‐Desmethylcarvedilol	C23H24N2O4	1.166	ESI‐	HMDB0013949	↓ ^∗^	↑#
24	11‐Beta_21‐dihydroxy‐5‐beta‐pregnane‐3_20‐dione	C21H32O4	7.99	ESI‐	HMDB0006757	↓ ^∗^	↑##
25	Linoleyl carnitine	C25H45NO4	8.158	ESI‐	HMDB0006469	↑ ^∗∗^	↓##
26	16‐Hydroxyhexadecanoic acid	C16H32O3	7.945	ESI‐	HMDB0006294	↑ ^∗^	↓#
27	1‐(5‐Phosphoribosyl) imidazole‐4‐acetate	C10H15N2O9P	1.192	ESI‐	HMDB0006032	↓ ^∗^	↑##
28	Lecithin	C42H80NO8P	7.782	ESI‐	HMDB0002159	↑ ^∗^	↓#
29	Butyryl‐L‐carnitine	C11H21NO4	3.048	ESI‐	HMDB0002013	↑ ^∗^	↓#
30	Thymidine5’‐monophosphate	C10H15N2O8P	1.154	ESI‐	HMDB0001227	↓ ^∗^	↑#
31	N‐Acetylaspartylglutamicacid	C11H16N2O8	1.255	ESI‐	HMDB0001067	↓ ^∗^	↑##
32	Hypotaurine	C2H7NO2S	0.786	ESI‐	HMDB0000965	↑ ^∗∗^	↓#
33	Succinyladenosine	C14H17N5O8	0.795	ESI‐	HMDB0000912	↑ ^∗∗^	↓##
34	Suberic acid	C8H14O4	4.013	ESI‐	HMDB0000893	↑ ^∗^	↓##
35	Propionylcarnitine	C10H19NO4	1.244	ESI‐	HMDB0000824	↑ ^∗^	↓##
36	Azelaic acid	C9 H16O4	4.51	ESI‐	HMDB0000784	↑ ^∗∗^	↓##
37	n‐Heptanoic acid	C7H14O2	3.972	ESI‐	HMDB0000666	↑ ^∗^	↓#
38	Decanoylcarnitine	C17H33NO4	8.939	ESI‐	HMDB0000651	↑ ^∗^	↓#
39	Uridine	C9H12N2O6	1.143	ESI‐	HMDB0000296	↓ ^∗^	↑#
40	Hypoxanthine	C5H4N4O	1.147	ESI‐	HMDB0000157	↓ ^∗^	↑#

*Note:* Compared to the control group, the model group was significantly increased/decreased ( ^∗^ *p* < 0.05,  ^∗∗^ *p* < 0.01); compared to the model group, the HZQYF‐M group was significantly increased/decreased (^#^ *p* < 0.05, ^##^ *p* < 0.01).

### 3.10. HZQYF Regulated the Abnormalities of Metabolites in Stool of Rat

To reveal the metabolic changes in stool samples of rat, we analyzed stool samples from three groups of rats (*n* = 10), control group, model group, and HZQYF‐M group, using LC‐MS/MS. A total of 10,699 metabolites were identified in the stool samples. The three groups of rats were separated by PLS‐DA (Figure 6A) and OPLS‐DA (Figure 6B,D) and clustered in each group, which showed significant differences among the three groups of rats. The R2 and Q2 of the OPLS‐DA scoring analysis verified a high level of predictability and good reliability (Figure 6C,E). Differential metabolites were identified by screening (VIP > 1.0, *p*  < 0.05). A total of 39 common metabolites were obtained from the three groups (Table 2). The apparent changes in metabolites in the three groups were compared by heat map analysis (Figure 6F). There was a significant increase in 10 metabolites and a decrease in 29 metabolites in the Model group compared to the control group. The expression of these metabolites was reversed after oral administration of HZQYF. The differentially expressed metabolites were further enriched using the MetaboAnalyst open database to generate KEGG metabolic pathways. Four different metabolic pathways were significantly enriched, including phenylalanine metabolism, steroid biosynthesis, gastric ulcer disease serine metabolism, and steroid hormone biosynthesis (Figure 6G). HMDB0012275 was determined to be involved in phenylalanine metabolism. HMDB0001181 was involved in steroid biosynthesis. HMDB0000034 was primarily involved in gastric ulcer disease serine metabolism. HMDB000183 was identified as a metabolic pathway involved in the metabolic pathway of KEGG. HMDB0001830 is primarily involved in steroid hormone biosynthesis. Notably, phenylalanine metabolism (*p* < 0.0208) was selected as the major metabolic pathway. As shown in Figure 6H–K, compared to the control group, the levels of HMDB0001181 and HMDB0000034 were elevated in the rats of the model group, while the levels of HMDB001275 and HMDB0001830 were decreased. Surprisingly, the expression trends of the above metabolisms were strongly reversed after administration of HZQYF, especially HMDB0012275, HMDB0001181, and HMDB0000034 (*p* < 0.05). In summary, HZQYF can effectively restore the metabolites in rat stool, thus contributing to the repair of gastric ulcers.

Figure 6Effect of HZQYF on fecal metabolic dysregulation in rat (*n* = 10). PLS‐DA (A) and OPLS‐DA (B, D) analysis score plots of metabolic profiles in the ion mode. (C, E) Cross‐validation results in ion mode. (F) Heat map showing differences in metabolite abundance. (G) KEGG pathway analysis of differential metabolites in the three groups. (H–K) Abundance comparison of key metabolites among the five groups based on KEGG results. Compared with control group,  ^∗^ *p* < 0.05,  ^∗∗^ *p* < 0.01; compared with the model group, ^#^ *p* < 0.05, ^##^ *p* < 0.01.

**Figure:**
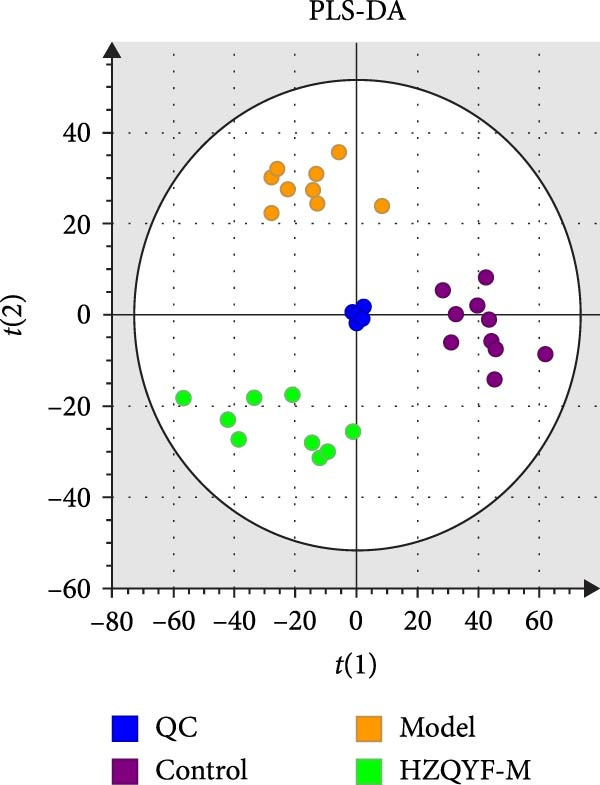
(A)

**Figure:**
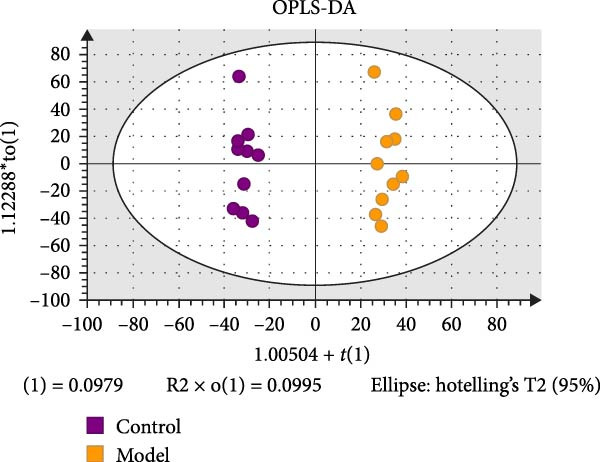
(B)

**Figure:**
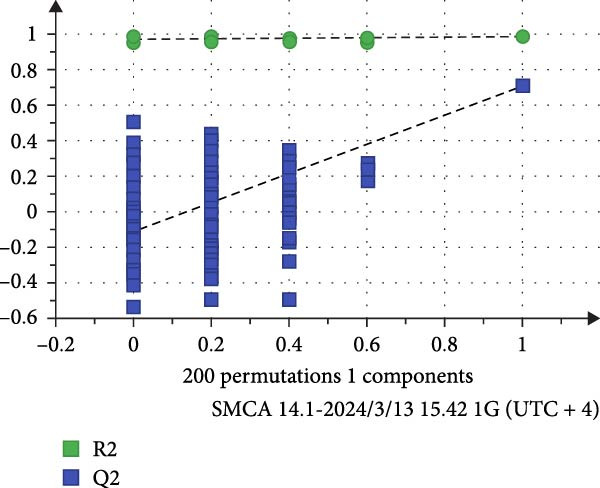
(C)

**Figure:**
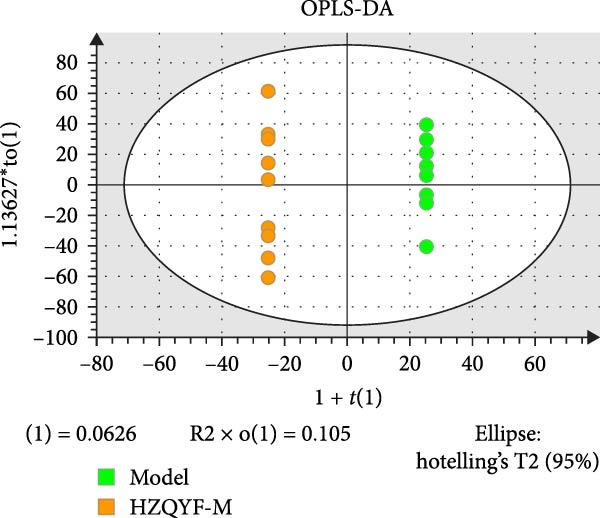
(D)

**Figure:**
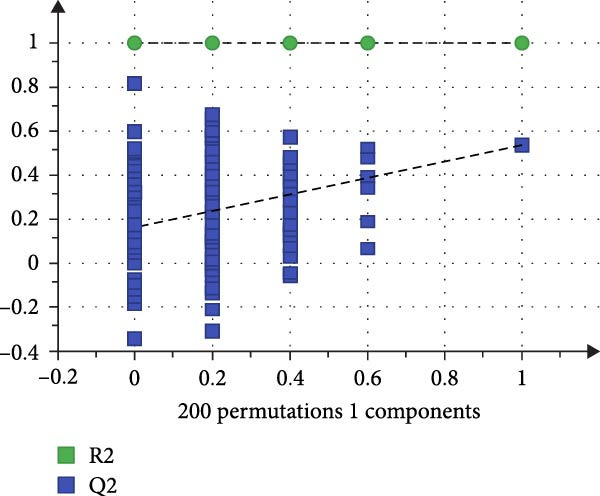
(E)

**Figure:**
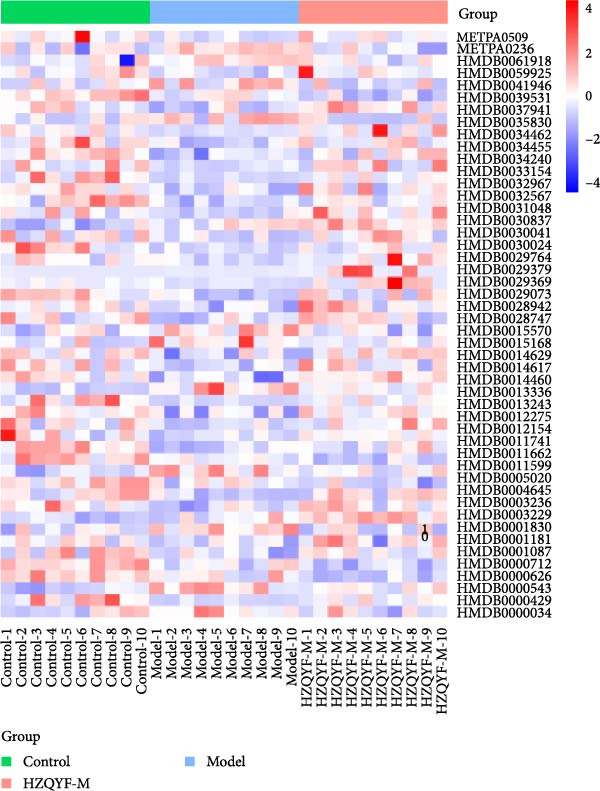
(F)

**Figure:**
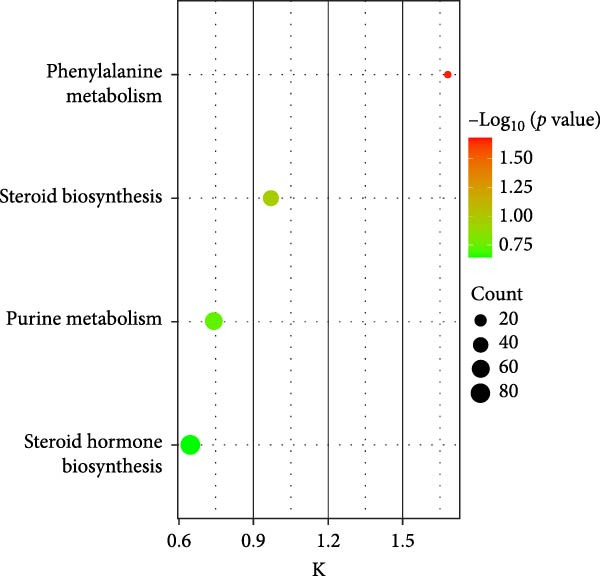
(G)

**Figure:**
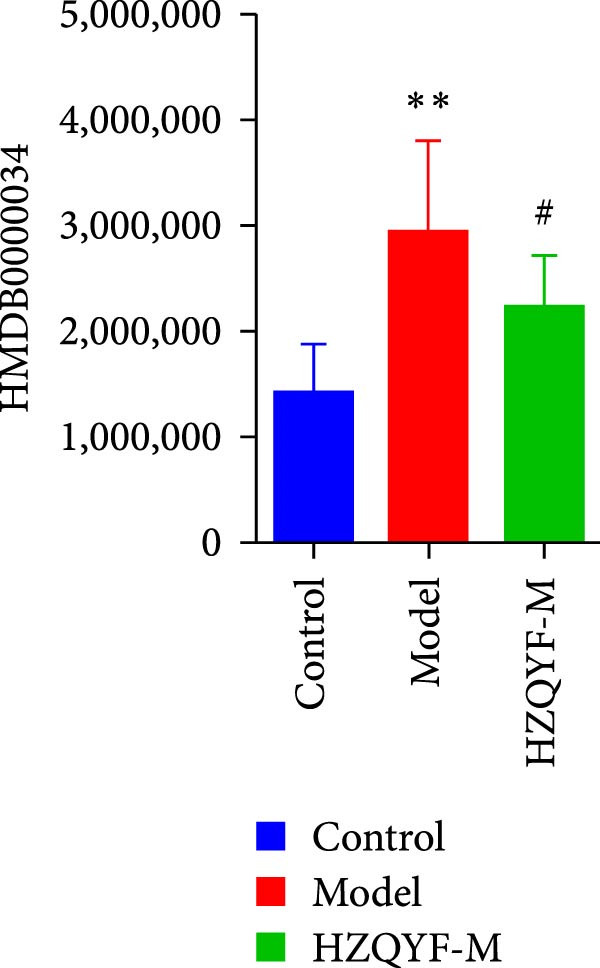
(H)

**Figure:**
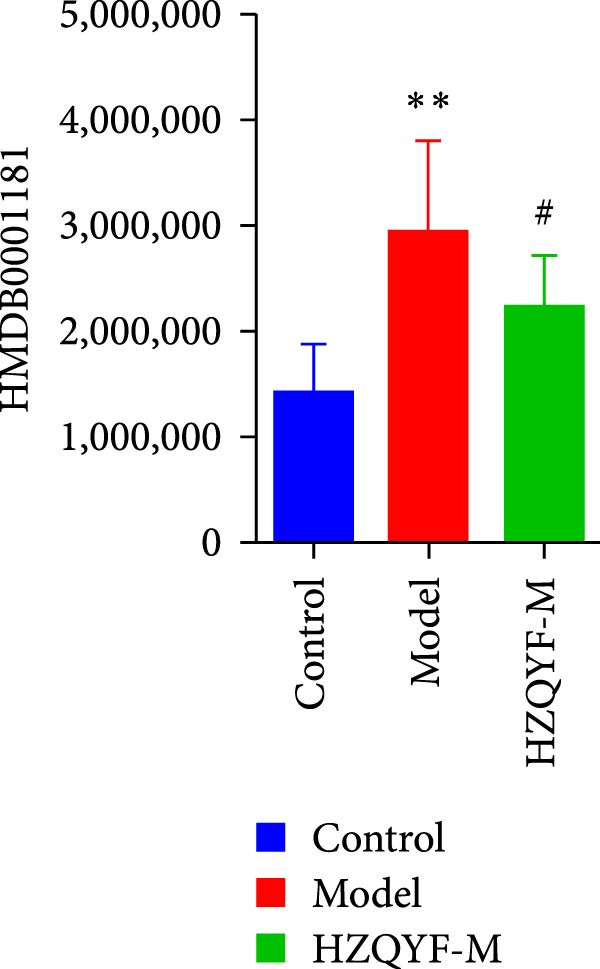
(I)

**Figure:**
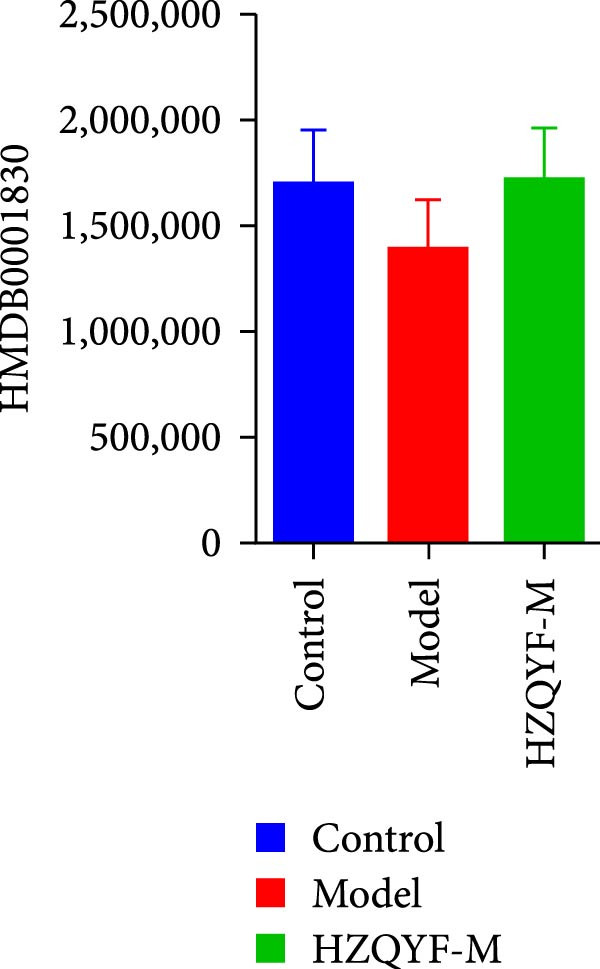
(J)

**Figure:**
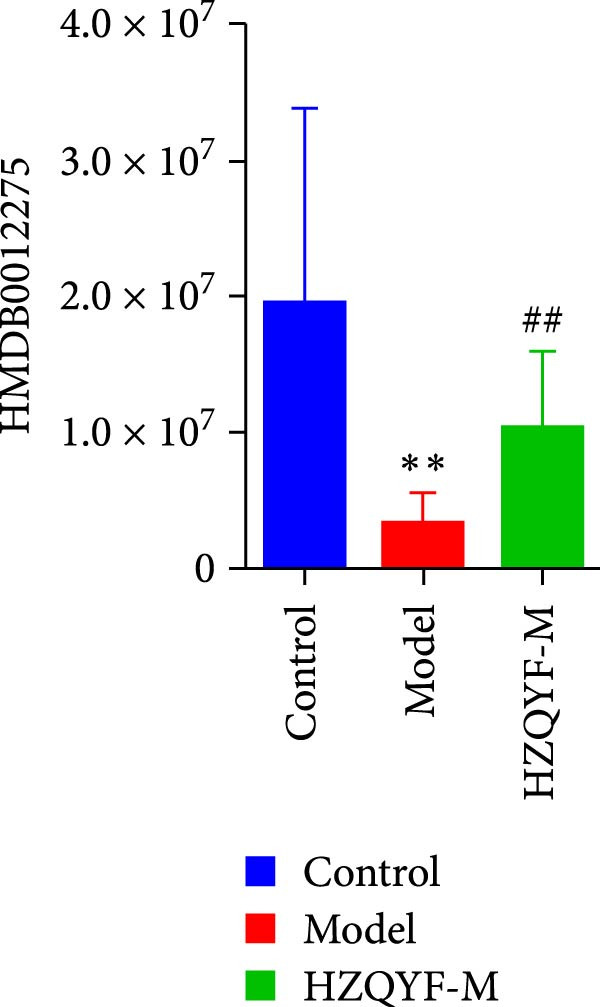
(K)

**Table 2:** Potential biomarkers in stool of rats with gastric ulcer treated with HZQYF.

Number	Name	Formula	RT (min)	Ion mode	HMDB	Model vs. control	HZQYF‐M vs. model
1	(R)‐3‐((R)‐3‐Hydroxybutanoyloxy) butanoate	C8H14O5	3.474	ESI‐	METPA0509	↓ ^∗∗^	↑#
2	2‐Aminophenol	C6H7NO	1.301	ESI‐	METPA0236	↑ ^∗^	↓#
3	2‐Oxindole	C8H7NO	2.137	ESI‐	HMDB0061918	↑ ^∗^	↓#
4	3‐BHA	C11H16O2	11.773	ESI‐	HMDB0059925	↓ ^∗^	↑#
5	N‐Nitrosoproline	C5H8N2O3	0.867	ESI‐	HMDB0041946	↑ ^∗^	↓##
6	Ergosterol peroxide	C28H44O3	11.184	ESI‐	HMDB0037941	↓ ^∗∗^	↑#
7	Genipin	C11H14O5	3.386	ESI‐	HMDB0035830	↑ ^∗^	↓#
8	Heptylparaben	C14H20O3	5.046	ESI‐	HMDB0034462	↓ ^∗∗^	↑#
9	Cyclopentadecanolide	C15H28O2	9.634	ESI‐	HMDB0034455	↓ ^∗∗^	↑##
10	Cyclohexapyrazine	C8H10N2	2.897	ESI‐	HMDB0033154	↓ ^∗^	↑#
11	2,4‐Dimethyl‐1 H‐indole	C10H11N	3.622	ESI‐	HMDB0032967	↓ ^∗^	↑##
12	Hexylresorcinol	C12H18O2	6.983	ESI‐	HMDB0032567	↓ ^∗^	↑##
13	Avocadyne 1‐acetate	C19H34O4	9.829	ESI‐	HMDB0031048	↓ ^∗^	↑#
14	trans‐Anethole	C10H12O	7.016	ESI‐	HMDB0030837	↓ ^∗^	↑##
15	(25 S)‐5beta‐Spirostan‐3beta‐ol	C27H44O3	6.224	ESI‐	HMDB0030024	↓ ^∗^	↑#
16	2‐Hydroxypropyl stearate	C21H42O3	10.248	ESI‐	HMDB0029764	↓ ^∗^	↑#
17	Sinapine	C16H23NO5	1.123	ESI‐	HMDB0029379	↓ ^∗^	↑#
18	thr‐tyr	C13H18N2O5	2.835	ESI‐	HMDB0029073	↓ ^∗^	↑#
19	Leu‐Val	C11H22N2O3	0.983	ESI‐	HMDB0028942	↓ ^∗∗^	↑#
20	asp‐arg	C10H19N5O5	0.897	ESI‐	HMDB0028747	↓ ^∗∗^	↑#
21	Fusidic acid	C31H48O6	9.128	ESI‐	HMDB0015570	↓ ^∗^	↑#
22	Cerulenin	C12H17NO3	4.844	ESI‐	HMDB0015168	↑ ^∗^	↓#
23	Nabilone	C24H36O3	9.27	ESI‐	HMDB0014629	↑ ^∗^	↓#
24	Methohexital	C14H18N2O3	3.807	ESI‐	HMDB0014617	↓ ^∗∗^	↑##
25	Zolmitriptan	C16H21N3O2	4.256	ESI‐	HMDB0014460	↓ ^∗∗^	↑#
26	3‐hydroxyhexadecanoylcarnitine	C23H45NO5	5.754	ESI‐	HMDB0013336	↓ ^∗∗^	↑#
27	leu‐phe	C15H22N2O3	3.989	ESI‐	HMDB0013243	↑ ^∗∗^	↓#
28	Phenethylamine	C8H11N	3.618	ESI‐	HMDB0012275	↓ ^∗∗^	↑##
29	3‐Dehydrocarnitine	C7H13NO3	1.504	ESI‐	HMDB0012154	↓ ^∗∗^	↑##
30	Gamma‐L‐glutamyl‐L‐tyrosine	C14H18N2O6	2.954	ESI‐	HMDB0011741	↓ ^∗^	↑##
31	3‐Beta‐hydroxy‐4beta‐methyl‐5alpha‐cholest‐7‐ene‐4alpha‐carboxylate	C29H48O3	9.809	ESI‐	HMDB0011662	↓ ^∗^	↑#
32	1‐Methyladenine	C6H7N5	2.866	ESI‐	HMDB0011599	↓ ^∗^	↑#
33	GSNO	C10H16N4O7S	2.893	ESI‐	HMDB0004645	↑ ^∗^	↓#
34	Palmitoleic acid	C16H30O2	9.814	ESI‐	HMDB0003229	↓ ^∗∗^	↑##
35	Hydroxyprogesterone caproate	C27H40O4	10.232	ESI‐	HMDB0001830	↓ ^∗∗^	↑#
36	5‐Methylthio‐D‐ribose	C6H12O4S	1.382	ESI‐	HMDB0001087	↑ ^∗^	↓#
37	Hexadecanedioic acid mono‐L‐carnitine ester	C23H43NO6	5.756	ESI‐	HMDB0000712	↓ ^∗^	↑#
38	Deoxycholic acid	C24H40O4	7.469	ESI‐	HMDB0000626	↓ ^∗^	↑##
39	Adenine	C5H5N5	3.12	ESI‐	HMDB0000034	↑ ^∗∗^	↓##

*Note:* Compared to the control group, the model group was significantly increased/decreased ( ^∗^ *p* < 0.05,  ^∗∗^ *p* < 0.01); compared to the model group, the HZQYF‐M group was significantly increased/decreased (^#^ *p* < 0.05, ^##^ *p* < 0.01).

### 3.11. HZQYF Down‐Regulates the MAPK Pathway

**Figure 7:**
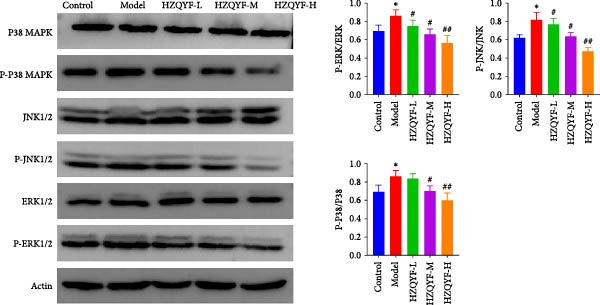
HZQYF regulates the expression of MAPK pathway related proteins in gastric ulcer rats (*n* = 3). The results were mean ± SD, *n* = 3. Compared with control group,  ^∗^ *p* < 0.05,  ^∗∗^ *p* < 0.01; compared with the model group, ^#^ *p* < 0.05, ^##^ *p* < 0.01.

Western blotting analysis was employed to assess the expression levels of key proteins involved in the study. The findings are presented in Figure 7. There was a significant upregulation of P‐P38MAPK, P‐JNK1/2, and ERK1/2 in the model group when compared to the control group (*p* < 0.05). In contrast, treatment with HZQYF resulted in a reversal of this upregulation in the HZQYF group, as evidenced by a significant decrease in the expression levels of these proteins (*p* < 0.05). Collectively, these results suggest that HZQYF mitigates gastric tissue inflammation and systemic inflammatory responses by suppressing the activation of the MAPK signaling pathway in rats.

## 4. Discussion

HZQYF is composed of *Terminalia chebula Retz*, *Ficus hirta* Vahl, and *Syzygium aromaticum* (*L*.) *Merr. & L.M.Perry*, their clinical dosages are 18, 9, and 3 g, respectively. After water extraction and drying, the yield of HZQYF was 26.42%. The human clinical dose (HCD) of HZQYF is 7.93 g/kg. According to the formula: rat dose = HCD × human Km/rat Km. The clinically equivalent dose to rats was calculated to be approximately 0.71 g/kg. Therefore, three different doses of HZQYF were chosen: low dose (0.36 g/kg), medium dose (0.71 g/kg), and high dose (1.42 g/kg), which were, respectively, equivalent to 0.5x, 1x, and 2x the clinical equivalent dose.

Acetic acid cauterizing gastric ulcer modeling is based on the damaging effect of acetic acid on gastric mucosa, which on the one hand produces inflammatory factors, on the other hand can produce a large number of oxygen free radicals, causing lipid peroxidation of mucosal cells, resulting in obstruction of mucosal blood supply, and further aggravating the formation of ulcer [32, 33]. SOD is the first line of defense in the biological body to automatically remove oxygen free radicals [34]. It has a super antioxidant effect, which can interrupt the chain reaction of oxygen free radicals and protect cells from damage. The level of its activity also indirectly reflects the ability of the body to remove oxygen free radicals. MDA is the degradation product of lipid peroxides in the body, and its content is an important indicator of oxygen free radical metabolism in the body, which can indirectly reflect the degree of cell damage [35, 36]. In this study, it was found that compared with the model group, the ulcer area in the administration group was significantly reduced, especially in the HZQYF‐M group (Figure 3A). Compared with the control group, the inflammatory factors (IL‐6, IL‐1β, and TNF‐α) in the model group were significantly increased and decreased to varying degrees after administration (Figure 3). At the same time, studies using network pharmacology to treat gastric ulcers have found that HZQYF can regulate MAPK pathway, and western blotting has proved that inflammatory response can be reduced by reducing P‐P38, P‐Erk1/2, and P‐JNK1/2protein.

VEGF is a glycoprotein that acts specifically on vascular endothelial cells and can directly and indirectly promote angiogenesis [37]. Research indicates that VEGF significantly contributes to the healing of ulcers. In addition to promoting angiogenesis, epithelial and granulation tissue hyperplasia, VEGF also promotes gastric mucosal epithelial hyperplasia and maintains mucosal integrity. TFF2, one of the subtypes of TFFs, can enhance the defense ability of gastric mucosa and promote the rapid repair of damaged mucosa [38]. PGE2 is an important gastric mucosal defense factor [39, 40]. EGF is a kind of endogenous protective substance that is not absorbed by the intestinal tract, can resist the digestion of protease and can inhibit the secretion of gastric acid [41]. Endogenous EGF in gastric juice can maintain the integrity of gastric mucosa. The results of this study showed that compared with the blank group, the contents of TFF2 and PGE2 in serum gastric tissue of rats with acetic acid burning gastric ulcer were decreased, indicating that acetic acid modeling caused mucosal damage and inhibited repair factors. After treatment with HZQYF, the expression of TFF2 and bFGF in serum increased, indicating that HZQYF can promote the repair of injured tissues by increasing the content of TFF2 and PGE2 in serum. Similarly, VEGF and EGF also increased significantly in the administration group compared to the control group.

## 5. Conclusions

In this comprehensive study, HZQYF demonstrated a multifaceted therapeutic impact on gastric ulcers in rats. It effectively reduced the ulcer index, ameliorated inflammatory responses, and normalized metabolic irregularities within gastric tissues and fecal matter. Most notably, HZQYF exerted a dual regulatory effect on key biomarkers: significantly suppressing pro‐inflammatory cytokines such as IL‐6, IL‐1β, and TNF‐α, while concurrently enhancing the expression of VEGF, EGF, TFF2, PGE2, and the antioxidant enzyme SOD. This effect not only effectively suppressed inflammatory responses but also significantly promoted tissue repair and antioxidant defense mechanisms. Furthermore, research has demonstrated that HZQYF can downregulate the MAPK signaling pathway in rats—a crucial mechanism mediating cellular responses and tissue repair processes. Collectively, these findings indicate that HZQYF mitigates inflammation and lowers the ulcer index by rebalancing the metabolic profiles of gastric tissues and fecal samples, thereby promoting gastric health and healing.

## Ethics Statement

The animal experimental procedures were meticulously conducted in accordance with the ethical standards set forth in the Guide for the Care and Use of Laboratory Animals. These procedures were rigorously reviewed and granted approval by the Ethics Committee for Experimental Animals at the State Key Laboratory of Generic Manufacture Technology of Chinese Traditional Medicine, with the specific approval number being AN‐IACUC‐2023‐072.

## Disclosure

All authors participated in the writing of the article and approved the final version for submission.

## Conflicts of Interest

The authors declare no conflicts of interest.

## Author Contributions

Zhong Feng and Ruixia Wei wrote the manuscript and prepared figures. Zhong Feng and Ruixia Wei contributed equally to this work and should be considered co‐first authors.

## Funding

No funding was received.

## Supporting Information

Additional supporting information can be found online in the Supporting Information section.

## Supporting information


**Supporting Information 1** Table S1. Raw data for Figure 1: communication target information. Table S2. Raw data for Figure 1C: GO analysis data. Table S3. Raw data for Figure 1D: DAVID pathway data. Table S4. Raw data for Figure 3A (ulcer area). Table S5. Raw data for Figure 3B (IL‐6). Table S6. Raw data for Figure 3C (IL‐1β). Table S7. Raw data for Figure 3D (TNF‐α). Table S8. Raw data for Figure 4A (VEGF). Table S9. Raw data for Figure 4B (EGF). Table S10. Raw data for Figure 4C (TFF2). Table S11. Raw data for Figure 4D (PGE2). Table S12. Raw data for Figure 4E (MDA). Table S13. Raw data for Figure 4F (SOD). Table S14. Raw data for Figure 5F: GO analysis data of rat gastric tissue. Table S15. Raw data for Figure 6F: GO analysis data of rat fecal. Figure S1. Raw data for Figure 7.

**Supporting Information 2** Data related to the metabolites according to the metabonomic analysis.

## Data Availability

The datasets generated and analyzed during the current study are available from the corresponding author upon reasonable request.
