# Supplementary material for: The Traditional Chinese Medicine Compound Hezi Qingyou Formula Controls the MAPK Signaling Pathway for Alleviating Gastric Ulcer Induced by Acetic Acid in Rats
Source: J Immunol Res. 2026 Jan 30;2026:2178079. doi: 10.1155/jimr/2178079 (PMC13140377; doi:10.1155/jimr/2178079)
Supplement: Supplementary file 1 — Supporting Information 1 Table S1. Raw data for Figure 1: communication target information. Table S2. Raw data for Figure 1C: GO analysis data. Table S3. Raw data for Figure 1D: DAVID pathway data. Table S4. Raw data for Figure 3A (ulcer area). Table S5. Raw data for Figure 3B (IL‐6). Table S6. Raw data for Figure 3C (IL‐1β). Table S7. Raw data for Figure 3D (TNF‐α). Table S8. Raw data for Figure 4A (VEGF). Table S9. Raw data for Figure 4B (EGF). Table S10. Raw data for Figure 4C (TFF2). Table S11. Raw data for Figure 4D (PGE2). Table S12. Raw data for Figure 4E (MDA). Table S13. Raw data for Figure 4F (SOD). Table S14. Raw data for Figure 5F: GO analysis data of rat gastric tissue. Table S15. Raw data for Figure 6F: GO analysis data of rat fecal. Figure S1. Raw data for Figure 7. [file JIMR-2026-2178079-s002.docx]

Supplementary Table 1 Raw data for Fig. 1 - Communication target information

| Communication target information | | | | | | | | | | | | | | | | |
| --- | --- | --- | --- | --- | --- | --- | --- | --- | --- | --- | --- | --- | --- | --- | --- | --- |
| GABRA2 | | CDK2 | | CCND1 | | CDK1 | | SLC6A4 | | TNF | | ITGAL | | PTGER1 | | PTGER4 |
| PTGER3 | | NOS2 | | PRKCA | | RORA | | PTGER2 | | G6PD | | GPBAR1 | | VDR | | POLB |
| CA9 | | LDHA | | TTR | | IGF1R | | SERPINE1 | | ESR2 | | BCL2L1 | | GPR35 | | TPMT |
| PTPN2 | | PTPN1 | | VEGFA | | FGF1 | | FGF2 | | HPSE | | HSP90AA1 | | ACHE | | NR3C1 |
| ALDH2 | | IL2 | | HSPA5 | | GAPDH | | MCL1 | | HSPA8 | | TOP1 | | LGALS3 | | PTGS2 |
| NQO2 | | ADRA2A | | XDH | | ALOX5 | | ERBB2 | | PDGFRB | | FLT4 | | EGFR | | AURKB |
| GSK3B | | SRC | | PTK2 | | KDR | | PLK1 | | CSNK2A1 | | MET | | TEK | | AKT1 |
| AURKA | | BRAF | | EPHB4 | | HSPA1A | | GSR | | ESR1 | | CYP1B1 | | ABCC1 | | ABCG2 |
| F2 | | MPO | | PIK3R1 | | DAPK1 | | MMP13 | | MMP3 | | MMP9 | | MMP2 | | CXCR1 |
| ABCB1 | | AXL | | AKR1A1 | | TOP2A | | SYK | | PIK3CG | | APEX1 | | CDK6 | | TERT |
| PLG | PARP1 | | MMP12 | | CYP1A2 | | NQO1 | | NFKB1 | | CDK4 | | CCNB1 | | CCNA2 | |

Supplementary Table 2 Raw data for Fig. 1C - GO analysis data

| GOterm | subgroup | Enrichment score |
| --- | --- | --- |
| negative regulation of synaptic transmission, dopaminergic | Biological process | 196.4949 |
| regulation of hepatocyte growth factor receptor signaling pathway | Biological process | 196.4949 |
| regulation of anaphase-promoting complex-dependent catabolic process | Biological process | 196.4949 |
| response to gold nanoparticle | Biological process | 196.4949 |
| regulation of endothelial cell apoptotic process | Biological process | 196.4949 |
| response to UV-A | Biological process | 196.4949 |
| aldehyde catabolic process | Biological process | 130.9966 |
| mononuclear cell migration | Biological process | 130.9966 |
| carboxylic acid transmembrane transport | Biological process | 130.9966 |
| regulation of endothelial cell chemotaxis to fibroblast growth factor | Biological process | 130.9966 |
| cyclin A2-CDK1 complex | Cellular component | 208.5556 |
| cyclin B1-CDK1 complex | Cellular component | 208.5556 |
| cyclin D1-CDK4 complex | Cellular component | 208.5556 |
| cyclin A2-CDK2 complex | Cellular component | 69.51852 |
| external side of apical plasma membrane | Cellular component | 69.51852 |
| chromosome passenger complex | Cellular component | 69.51852 |
| sorting endosome | Cellular component | 69.51852 |
| plasma membrane region | Cellular component | 52.13889 |
| Bcl-2 family protein complex | Cellular component | 52.13889 |
| phosphatidylinositol 3-kinase complex, class IA | Cellular component | 46.34568 |
| cyclin-dependent protein kinase activity | Molecular function | 191.5859 |
| prostaglandin E receptor activity | Molecular function | 153.2687 |
| NAD(P)H dehydrogenase (quinone) activity | Molecular function | 127.7239 |
| estrogen receptor activity | Molecular function | 95.79293 |
| carboxylic acid transmembrane transporter activity | Molecular function | 95.79293 |
| death receptor agonist activity | Molecular function | 95.79293 |
| nitric-oxide synthase regulator activity | Molecular function | 95.79293 |
| insulin binding | Molecular function | 76.63434 |
| ErbB-3 class receptor binding | Molecular function | 76.63434 |
| histone kinase activity | Molecular function | 76.63434 |

Supplementary Table 3 Raw data for Fig. 1D - DAVID pathway data

| Description | GeneRatio | pvalue | GeneID | Count |
| --- | --- | --- | --- | --- |
| Pathways in cancer | 0.07 | 1.89 | PTGER4/ GSK3B/ PTGER1/ FLT4/ PTGER2/ PTGER3/ PIK3R1/ FGF1/ PTGS2/ FGF2/ EGFR/ IGF1R/ CCND1/ TERT/ ERBB2/ AKT1/ PDGFRB/ NQO1/ HSP90AA1/ NOS2/ DAPK1/ MMP2/ PRKCA/ BRAF/ F2/ MMP9/ ESR1/ IL2/ PTK2/ ESR2/ NFKB1/ VEGFA/ CCNA2/ CDK6/ CDK4/ CDK2/ MET/ BCL2L1 | 38 |
| EGFR tyrosine kinase inhibitor resistance | 0.20 | 2.88 | PDGFRB/ GSK3B/ SRC/ BRAF/ PRKCA/ PIK3R1/ FGF2/ EGFR/ IGF1R/ VEGFA/ AXL/ ERBB2/ KDR/ AKT1/ MET/ BCL2L1 | 16 |
| PI3K-Akt signaling pathway | 0.07 | 3.06 | GSK3B/ FLT4/ PIK3R1/ FGF1/ FGF2/ EGFR/ PIK3CG/ IGF1R/ CCND1/ ERBB2/ KDR/ AKT1/ MCL1/ PDGFRB/ HSP90AA1/ SYK/ PRKCA/ IL2/ PTK2/ NFKB1/ VEGFA/ CDK6/ CDK4/ CDK2/ TEK/ MET/ BCL2L1 | 27 |
| Proteoglycans in cancer | 0.09 | 4.07 | SRC/ MMP2/ BRAF/ PRKCA/ PIK3R1/ FGF2/ ESR1/ TNF/ MMP9/ EGFR/ PTK2/ IGF1R/ VEGFA/ CCND1/ ERBB2/ KDR/ AKT1/ HPSE/ MET | 19 |
| Prostate cancer | 0.14 | 2.44 | PDGFRB/ GSK3B/ HSP90AA1/ MMP3/ BRAF/ PIK3R1/ MMP9/ EGFR/ NFKB1/ IGF1R/ CCND1/ ERBB2/ CDK2/ AKT1 | 14 |
| Endocrine resistance | 0.14 | 2.78 | SRC/ MMP2/ BRAF/ PIK3R1/ ESR1/ MMP9/ EGFR/ PTK2/ ESR2/ IGF1R/ CCND1/ CDK4/ ERBB2/ AKT1 | 14 |
| Human cytomegalovirus infection | 0.08 | 1.88 | PTGER4/ GSK3B/ PTGER1/ SRC/ PTGER2/ PTGER3/ PRKCA/ PIK3R1/ PTGS2/ TNF/ EGFR/ PTK2/ NFKB1/ VEGFA/ CDK6/ CCND1/ CDK4/ AKT1 | 18 |
| Melanoma | 0.17 | 2.15 | PDGFRB/ CDK6/ CCND1/ CDK4/ AKT1/ BRAF/ PIK3R1/ FGF1/ FGF2/ MET/ EGFR/ IGF1R | 12 |
| Bladder cancer | 0.24 | 3.43 | CCND1/ SRC/ CDK4/ DAPK1/ MMP2/ ERBB2/ BRAF/ MMP9/ EGFR/ VEGFA | 10 |
| Breast cancer | 0.10 | 4.25 | GSK3B/ FLT4/ BRAF/ PIK3R1/ FGF1/ FGF2/ ESR1/ EGFR/ ESR2/ IGF1R/ CDK6/ CCND1/ CDK4/ ERBB2/ AKT1 | 15 |
| HIF-1 signaling pathway | 0.12 | 1.55 | NOS2/ SERPINE1/ PRKCA/ PIK3R1/ EGFR/ NFKB1/ IGF1R/ VEGFA/ LDHA/ ERBB2/ AKT1/ TEK/ GAPDH | 13 |
| Focal adhesion | 0.07 | 3.38 | PDGFRB/ GSK3B/ SRC/ FLT4/ BRAF/ PRKCA/ PIK3R1/ EGFR/ PTK2/ IGF1R/ VEGFA/ CCND1/ ERBB2/ KDR/ AKT1/ MET | 16 |
| Rap1 signaling pathway | 0.07 | 5.39 | PDGFRB/ SRC/ FLT4/ BRAF/ PRKCA/ PIK3R1/ ITGAL/ FGF1/ FGF2/ EGFR/ IGF1R/ VEGFA/ KDR/ AKT1/ TEK/ MET | 16 |
| Pancreatic cancer | 0.14 | 6.76 | CDK6/ CCND1/ CDK4/ ERBB2/ AKT1/ BRAF/ PIK3R1/ EGFR/ NFKB1/ BCL2L1/ VEGFA | 11 |
| MAPK signaling pathway | 0.06 | 1.63 | PDGFRB/ HSPA8/ FLT4/ BRAF/ PRKCA/ FGF1/ FGF2/ TNF/ EGFR/ NFKB1/ IGF1R/ VEGFA/ ERBB2/ KDR/ AKT1/ TEK/ MET/ HSPA1A | 18 |
| Measles | 0.09 | 2.34 | HSPA8/ GSK3B/ CSNK2A1/ PIK3R1/ IL2/ NFKB1/ CDK6/ CCND1/ CDK4/ CDK2/ AKT1/ HSPA1A/ BCL2L1 | 13 |
| Hepatocellular carcinoma | 0.08 | 2.39 | NQO1/ GSK3B/ BRAF/ PRKCA/ PIK3R1/ EGFR/ IGF1R/ CDK6/ TERT/ CCND1/ CDK4/ AKT1/ MET/ BCL2L1 | 14 |
| Small cell lung cancer | 0.12 | 4.43 | CDK6/ CCND1/ NOS2/ CDK4/ CDK2/ AKT1/ PIK3R1/ PTGS2/ PTK2/ NFKB1/ BCL2L1 | 11 |
| Chemical carcinogenesis - receptor activation | 0.07 | 4.97 | HSP90AA1/ SRC/ VDR/ PRKCA/ PIK3R1/ FGF2/ ESR1/ EGFR/ ESR2/ NFKB1/ VEGFA/ CCND1/ CYP1A2/ CYP1B1/ AKT1 | 15 |
| Gastric cancer | 0.08 | 5.54 | GSK3B/ ABCB1/ BRAF/ PIK3R1/ FGF1/ FGF2/ EGFR/ TERT/ CCND1/ ERBB2/ CDK2/ AKT1/ MET | 13 |

Supplementary Table 4 Raw data for Fig. 3A (ulcer area)

| Control(mm^2^) | Model(mm^2^) | HZQYF-L(mm^2^) | HZQYF-M(mm^2^) | HZQYF-H(mm^2^) |
| --- | --- | --- | --- | --- |
| 13.26 | 10.79 | 7.64 | 12.23 | 6.69 |
| 9.58 | 8.81 | 8.97 | 9.31 | 8.93 |
| 20.78 | 4.93 | 11.31 | 8.67 | 7.55 |
| 12.91 | 7.85 | 12.35 | 13.59 | 7.40 |
| 12.06 | 9.095 | 11.82 | 11.88 | 8.24 |
| 8.99 | 7.39 | 13.04 | 7.41 | 7.74 |
| 7.22 | 12.09 | 13.71 | 10.09 | 5.44 |
| 10.60 | 11.60 | 9.86 | 11.10 | 11.73 |
| 17.80 | 8.63 | 7.94 | 12.82 | 7.87 |
| 11.11 | 11.62 | 12.12 | 11.93 | 10.04 |

Supplementary Table 5 Raw data for Fig. 3B (IL-6)

| Control(pg/mL) | Model(pg/mL) | HZQYF-L(pg/mL) | HZQYF-M(pg/mL) | HZQYF-H(pg/mL) |
| --- | --- | --- | --- | --- |
| 10.18 | 15.07 | 13.29 | 13.62 | 12.4 |
| 14.62 | 14.84 | 12.51 | 13.73 | 14.95 |
| 14.96 | 15.14 | 14.18 | 12.62 | 11.62 |
| 13.62 | 17.18 | 15.07 | 12.4 | 14.29 |
| 12.62 | 14.96 | 12.62 | 11.07 | 13.28 |
| 12.07 | 15.4 | 13.29 | 11.84 | 11.91 |
| 13.98 | 12.09 | 11.07 | 15.78 | 13.78 |
| 12.67 | 14.08 | 15.33 | 12.89 | 14.92 |
| 14.05 | 16.52 | 15.69 | 14.62 | 12.69 |
| 13.27 | 14.83 | 12.36 | 14.59 | 14.75 |

Supplementary Table 6 Raw data for Fig. 3C (IL-1β)

| Control(pg/mL) | Model(pg/mL) | HZQYF-L(pg/mL) | HZQYF-M(pg/mL) | HZQYF-H(pg/mL) |
| --- | --- | --- | --- | --- |
| 1.49 | 1.6 | 1.36 | 1.44 | 1.33 |
| 1.26 | 1.89 | 1.62 | 1.69 | 1.61 |
| 1.47 | 1.76 | 1.47 | 1.33 | 1.55 |
| 1.33 | 1.57 | 1.51 | 1.76 | 1.23 |
| 1.62 | 1.63 | 1.45 | 1.62 | 1.47 |
| 1.26 | 1.58 | 1.69 | 1.47 | 1.43 |
| 1.35 | 1.52 | 1.59 | 1.41 | 1.78 |
| 1.67 | 1.78 | 1.73 | 1.38 | 1.63 |
| 1.45 | 1.66 | 1.68 | 1.54 | 1.32 |
| 1.17 | 1.56 | 1.67 | 1.63 | 1.24 |

Supplementary Table 7 Raw data for Fig. 3D (TNF-α)

| Control(pg/mL) | Model(pg/mL) | HZQYF-L(pg/mL) | HZQYF-M(pg/mL) | HZQYF-H(pg/mL) |
| --- | --- | --- | --- | --- |
| 23.27 | 25.86 | 22.93 | 24.64 | 27.5 |
| 23.25 | 26.23 | 26.26 | 26.63 | 26.43 |
| 24.18 | 25.02 | 24.86 | 25.92 | 24.41 |
| 26.91 | 29.54 | 25.32 | 23.78 | 24.48 |
| 23.72 | 28.5 | 26.94 | 24.18 | 22.68 |
| 25.32 | 26.38 | 25.97 | 27.35 | 23.58 |
| 25.38 | 30.67 | 24.58 | 25.76 | 26.74 |
| 24.93 | 27.63 | 23.98 | 24.81 | 25.85 |
| 23.69 | 26.59 | 27.84 | 25.77 | 24.69 |
| 24.41 | 25.57 | 24.91 | 22.89 | 23.71 |

Supplementary Table 8 Raw data for Fig. 4A (VEGF)

| Control(pg/mL) | Model(pg/mL) | HZQYF-L(pg/mL) | HZQYF-M(pg/mL) | HZQYF-H(pg/mL) |
| --- | --- | --- | --- | --- |
| 42.75 | 54.53 | 49.09 | 55.83 | 52.57 |
| 41.66 | 54.72 | 53.92 | 52.21 | 51.47 |
| 40.27 | 32.79 | 50.03 | 54.47 | 51.12 |
| 38.27 | 53.95 | 50.10 | 35.46 | 46.92 |
| 37.60 | 54.57 | 35.14 | 51.46 | 31.81 |
| 42.47 | 53.25 | 56.53 | 55.79 | 32.23 |
| 32.85 | 57.62 | 35.22 | 56.84 | 24.56 |
| 40.49 | 48.68 | 38.99 | 49.66 | 40.47 |
| 27.95 | 49.45 | 36.66 | 54.96 | 52.16 |
| 45.12 | 41.69 | 47.65 | 46.35 | 34.62 |

Supplementary Table 9 Raw data for Fig. 4B (EGF)

| Control(pg/mL) | Model(pg/mL) | HZQYF-L(pg/mL) | HZQYF-M(pg/mL) | HZQYF-H(pg/mL) |
| --- | --- | --- | --- | --- |
| 204.35 | 239.48 | 275.70 | 403.54 | 270.57 |
| 164.62 | 241.92 | 195.70 | 267.05 | 240.29 |
| 198.95 | 173.54 | 319.49 | 291.11 | 283.54 |
| 226.78 | 207.59 | 232.73 | 401.13 | 415.16 |
| 276.78 | 217.32 | 313.81 | 244.89 | 292.46 |
| 214.89 | 250.57 | 372.73 | 211.38 | 314.62 |
| 214.56 | 240.38 | 366.96 | 245.14 | 184.66 |
| 255.79 | 202.98 | 184.07 | 354.65 | 284.14 |
| 201.46 | 186.09 | 324.34 | 361.06 | 351.23 |
| 193.52 | 166.71 | 309.16 | 231.56 | 166.56 |

Supplementary Table 10 Raw data for Fig. 4C (TFF2)

| Control(pg/mL) | Model(pg/mL) | HZQYF-L(pg/mL) | HZQYF-M(pg/mL) | HZQYF-H(pg/mL) |
| --- | --- | --- | --- | --- |
| 2.21 | 1.82 | 1.76 | 1.94 | 1.85 |
| 2.30 | 1.84 | 1.58 | 2.05 | 1.63 |
| 2.27 | 1.69 | 1.78 | 1.75 | 1.77 |
| 2.16 | 1.47 | 1.49 | 1.74 | 1.88 |
| 2.19 | 1.85 | 1.79 | 1.69 | 1.84 |
| 1.54 | 1.60 | 1.56 | 2.13 | 1.56 |
| 1.79 | 1.85 | 1.68 | 2.05 | 1.59 |
| 2.37 | 1.65 | 1.46 | 1.66 | 1.37 |
| 1.82 | 1.51 | 1.60 | 2.25 | 1.88 |
| 2.18 | 1.89 | 1.85 | 2.15 | 1.84 |

Supplementary Table 11 Raw data for Fig. 4D (PGE2)

| Control(pg/mL) | Model(pg/mL) | HZQYF-L(pg/mL) | HZQYF-M(pg/mL) | HZQYF-H(pg/mL) |
| --- | --- | --- | --- | --- |
| 28.62 | 21.77 | 29.37 | 35.27 | 53.82 |
| 24.93 | 31.57 | 56.32 | 63.93 | 35.51 |
| 40.39 | 17.58 | 45.95 | 36.32 | 36.75 |
| 29.55 | 25.32 | 54.42 | 56.98 | 48.60 |
| 29.32 | 21.69 | 48.59 | 25.99 | 38.94 |
| 47.61 | 32.47 | 23.20 | 52.36 | 47.61 |
| 28.98 | 31.45 | 30.65 | 34.87 | 35.28 |
| 36.16 | 23.61 | 58.32 | 55.94 | 21.38 |
| 34.66 | 26.19 | 52.66 | 27.38 | 34.17 |
| 44.89 | 35.54 | 28.48 | 55.81 | 21.25 |

Supplementary Table 12 Raw data for Fig. 4E (MDA)

| Control(nmol/mL) | Model(nmol/mL) | HZQYF-L(nmol/mL) | HZQYF-M(nmol/mL) | HZQYF-H(nmol/mL) |
| --- | --- | --- | --- | --- |
| 0.62 | 1.41 | 0.98 | 0.83 | 0.89 |
| 0.67 | 0.93 | 1.39 | 0.82 | 1.19 |
| 1.19 | 0.78 | 1.19 | 1.14 | 0.72 |
| 1.03 | 0.79 | 0.72 | 0.88 | 0.667 |
| 0.62 | 0.78 | 0.77 | 0.77 | 0.62 |
| 0.61 | 1.24 | 1.24 | 0.72 | 0.61 |
| 0.75 | 1.14 | 0.84 | 0.64 | 0.73 |
| 0.68 | 1.24 | 0.85 | 0.72 | 0.84 |
| 0.66 | 0.88 | 0.73 | 0.65 | 1.04 |
| 0.79 | 0.87 | 0.93 | 0.62 | 0.74 |

Supplementary Table 13 Raw data for Fig. 4F (SOD)

| Control(U/mL) | Model(U/mL) | HZQYF-L(U/mL) | HZQYF-M(U/mL) | HZQYF-H(U/mL) |
| --- | --- | --- | --- | --- |
| 55.55 | 44.23 | 47.16 | 39.76 | 39.56 |
| 46.22 | 38.98 | 42.88 | 40.55 | 38.98 |
| 45.90 | 42.48 | 31.49 | 41.38 | 45.90 |
| 33.73 | 36.97 | 38.60 | 39.17 | 50.68 |
| 49.18 | 28.99 | 36.44 | 49.28 | 39.95 |
| 53.00 | 38.23 | 32.48 | 50.97 | 44.47 |
| 39.43 | 34.69 | 45.85 | 50.17 | 37.68 |
| 42.46 | 43.46 | 38.03 | 34.33 | 41.13 |
| 36.98 | 33.83 | 41.93 | 47.05 | 41.99 |
| 51.59 | 37.36 | 30.28 | 43.96 | 46.81 |

Supplementary Table 14 Raw data for Fig. 5F - GO analysis data of rat gastric tissue

| Tlotal | Hits | Expect | P value | Holm P |
| --- | --- | --- | --- | --- |
| Taurine and hypotaurine metabolism | 8 | 1 | 0.026 | 0.0258 |
| Lysine degradation | 30 | 1 | 0.0975 | 0.0939 |
| Pyrimidine metabolism | 39 | 1 | 0.127 | 0.121 |
| Drug metabolism - cytochrome P450 | 55 | 1 | 0.179 | 0.167 |
| Purine metabolism | 70 | 1 | 0.228 | 0.208 |

Supplementary Table 15 Raw data for Fig. 6F - GO analysis data of rat fecal

| Tlotal | Hits | Expect | P value | Holm P |
| --- | --- | --- | --- | --- |
| Phenylalanine metabolism | 8 | 1 | 0.0208 | 0.0207 |
| Steroid biosynthesis | 41 | 1 | 0.107 | 0.102 |
| Purine metabolism | 70 | 1 | 0.182 | 0.17 |
| Steroid hormone biosynthesis | 87 | 1 | 0.226 | 0.208 |

Repeat 1 Repeat2 Repeat 3


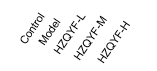

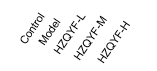

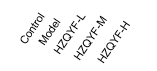


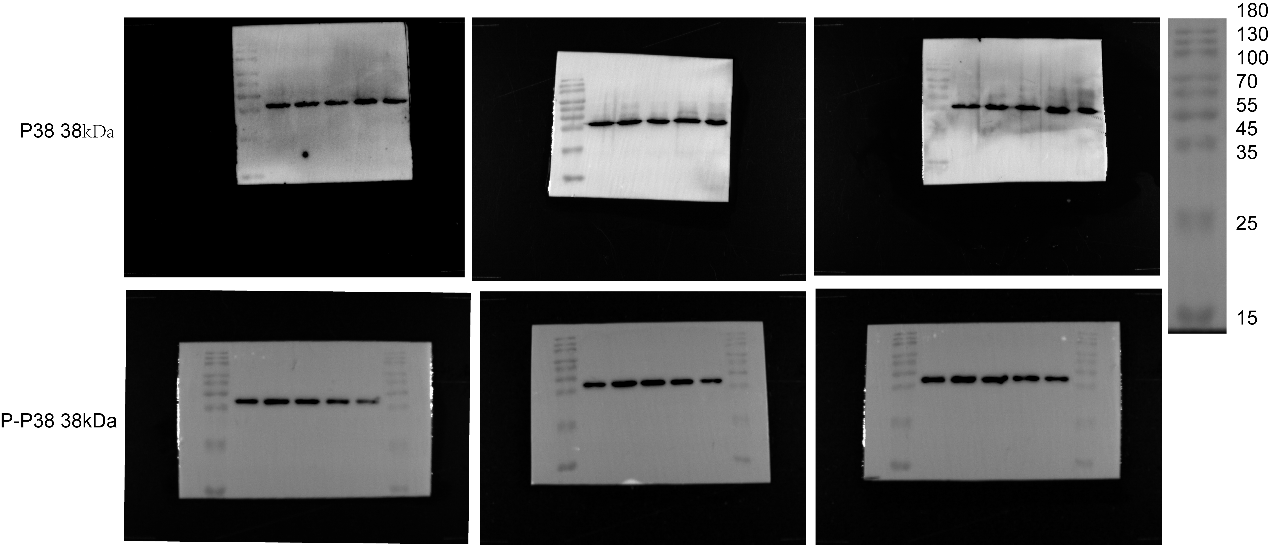


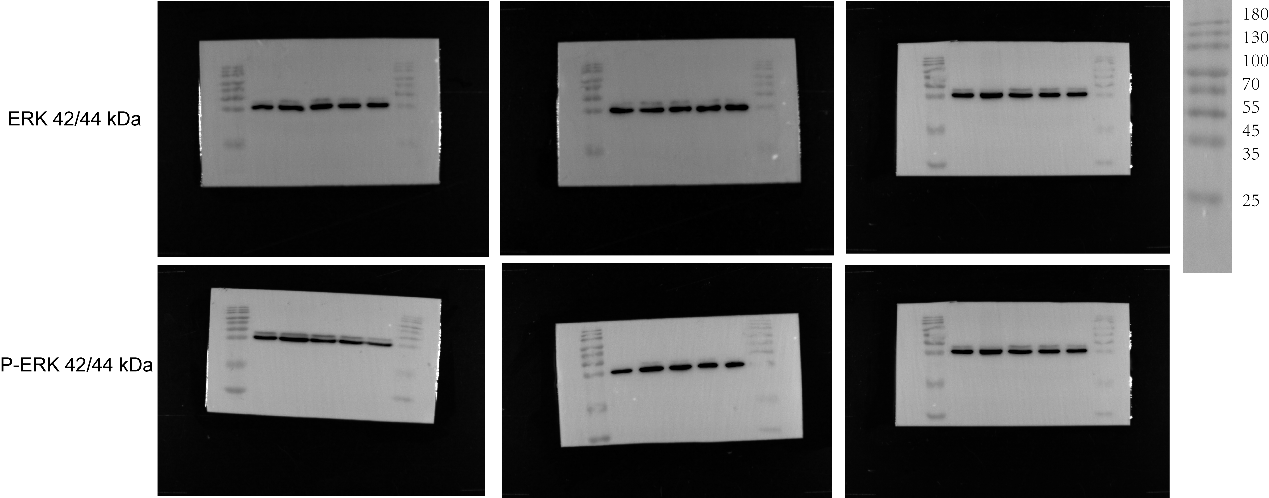


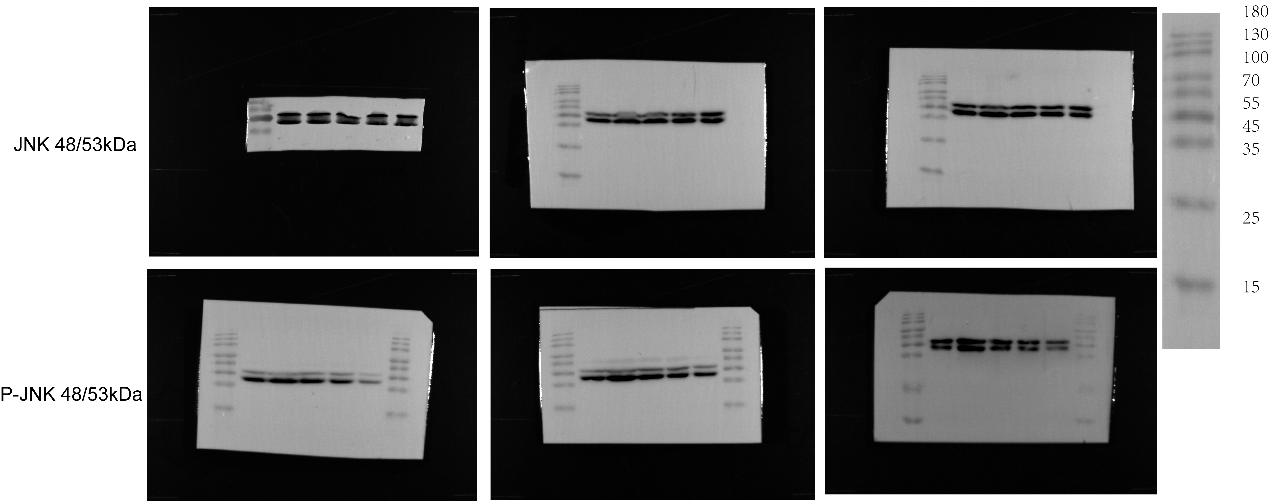


Repeat 1 Repeat2 Repeat 3


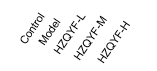

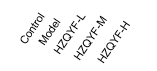

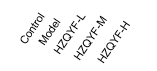


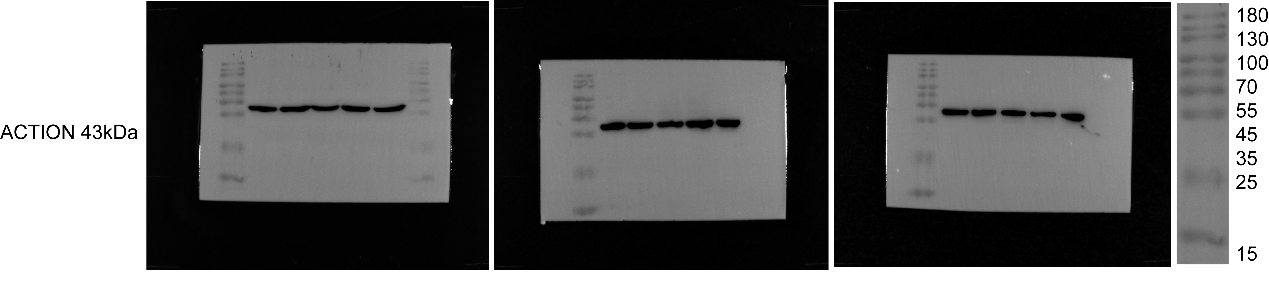


Supplementary Figure 1. Raw data for Fig. 7
